# Experimental and Theoretical Study of Kinetics of Bulk Crystallization in Poly(Chlorotrifluoroethylene)

**DOI:** 10.6028/jres.063A.005

**Published:** 1959-08-01

**Authors:** John D. Hoffman, James J. Weeks, W. M. Murphey

## Abstract

The rate of isothermal bulk crystallization of poly(chlorotrifluoroethylene), *T_m_*=221° C, was measured from 170° to 200° C. The intrinsic bulk crystallization, which accurately followed an *n* = 2 law, was shown to be a result of the injection of primary nuclei sporadically in time, with one-dimensional growth of centers derived from these nuclei. The crystallites are exceedingly small. The one-dimensional growth process was isolated by nucleating specimens with seed crystals, and its temperature-dependence determined between 191° and 205° C. The seed crystal isotherms followed an *n* = 1 law. The temperature coefficients of the rate of nucleation and the rate of growth were both strongly negative.

A theory of homogeneous nucleation that takes into account the segmental character of the polymer chains is developed in some detail. A cylindrical nucleus is assumed. In the temperature range near the melting point, region A, where the radius and length of the nucleus are unrestricted, the rate of nucleation is shown to be proportional to exp(−*α*/*T*^3^Δ*T*^2^). The nucleation rate is proportional to exp (−*β*/*T^2^*Δ*T*) in region B, which extends from somewhat below the melting point to considerably lower temperatures; the length of the nucleus has a constant value *l*_0_ in this region, but the radius is unrestricted. (In the above expressions, *α* and *β* are constants). Finally, at sufficiently low temperatures, region C is entered. Under certain circumstances, the rate of nucleation in region C will be extremely rapid, and correspond to a “nucleative collapse” of the supercooled liquid state. A calculation of the one-dimensional growth rate shows that it is proportional to exp(−*γ/T^2^*Δ*T*) where *β*=*γ*.

A careful analysis of the experimental data obtained between 170° and 200° C clearly showed that both the rate of nucleation and the rate of growth were proportional to exp(−*β*/*T*^2^Δ*T*), and not exp(−*α/T*^3^Δ*T*^2^). The primary nucleation event was thus of type B in this interval. A detailed analysis of the data is given, and surface free energies and the dimensions of the nuclei quoted. Quenching experiments, where the polymer was crystallized well below 170° C, gave a firm indication of the existence of region C.

An experimental study was made of the extremely slow crystallization process that prevailed when the degree of crystallinity became high. The onset of this stage of the crystallization was interpreted as being the result of a massive degree of impingement. This interpretation is justified by the calculations of Lauritzen, who has given a theory of impingements that predicts a pseudoequilibrium degree of crystallinity.

As indicated above, the growth process originating at homogeneous nuclei is not of a three-dimensional or spherulitic character in the region of study. Such stray spherulites as do appear in this region are shown to originate at heterogeneities. The possibility that the intrinsic growth process may become three-dimensional at crystallization temperatures sufficiently near *T_m_* is discussed.

## 1. Introduction

Poly(chlorotrifluoroethylene) is a linear homopolymer that possesses a strong tendency to crystallize when stored in the supercooled state. This paper is concerned with a detailed experimental and theoretical study of this phenomenon. Rate of crystallization studies were carried out on this polymer with three basic objectives in mind. In enumerating these objectives below, the opportunity is taken to touch briefly on the methods and reasoning used to attain them.

The first objective of the work was to determine the experimental conditions conducive to homogeneous nucleation, and to investigate the geometry of crystal growth under these conditions. The basic aim here was the uncovering of intrinsic properties of the polymer, as opposed to those resulting from interaction of the polymer with foreign bodies.

Overall (bulk) crystallization isotherms were obtained at various temperatures below the melting point by measuring the decrease of volume with time for samples of polymer that had been initially superheated far above the melting point. As expected from the work of Turnbull [1],^1^ the strong superheating greatly subdued the effect of embryos stabilized in pores or fissures in heterogeneities, and led to highly reproducible results. In as much as the relationship between volume and degree of crystallinity, *x*, is accurately known [2] for poly(chlorotrifluoroethylene), the isotherms were expressed as plots of *x* versus log *t*, where *t* was the time as measured from the inception of the experiment. In general, the free bulk growth rate may be represented as *x′* = *Zt^n^*, where *n* is a number depending on the type of nucleation and the geometry of growth, and *Z* is a rate constant. It was determined by a straightforward analysis of the data that the isotherms obtained with strong superheating corresponded to *x′* = *Z*_2_*t*^2^, i.e., to *n* = 2. This suggested that the crystallization was a result of one-dimensional growth of primary nuclei that were born at later and later dates. The alternative, and altogether less likely, explanation is that the crystallization was the result of two-dimensional growth of objects born at the same time. Any ambiguity in the result that the mode of growth was of a onedimensional character was removed by carrying out a crystallization initiated by seed crystals arising from a previous run where the free bulk growth rate was described by *n* = 2. It was determined that *n* = 1 when the crystallization was initiated by such seed crystals. The only physically reasonable interpretation for a free bulk growth rate where *n* = 1 is that the crystallization was due to one-dimensional growth of objects born at the same time. These experiments made it clear that the isotherms for which the *n* = 2 growth law was observed are in fact to be interpreted in terms of one-dimensional growth of objects born at later and later dates. The birth of objects at later and later dates may arise from either true homogeneous nucleation, or pseudohomogeneous nucleation, the latter being a result of nearly sporadic initiation on flat surfaces on heterogeneities (see below).

The fact that it can be demonstrated that the primary nuclei are born at later and later dates does not in itself necessarily mean that the nucleation is of a truly homogeneous character. Homogeneous nucleation refers to that process where crystallization centers are spontaneously formed at random positions in the pure mother phase by thermal fluctuations. Such a process is characterized by a rate of production of nuclei per unit volume of mother phase that is truly constant in time, except perhaps for a very short induction period. However, a process of a rather similar character can take place on flat surfaces on any heterogeneities that may exist in the system. In this case, fluctuations near flat surfaces that are wettable by the crystalline phase can cause nuclei to be born at a rate that decreases only rather slowly with time, so that the rate of injection may be very nearly constant. (This holds if the system has previously been superheated to a degree sufficient to melt out any embryos in fissures or pores in the heterogeneities that would otherwise act as centers of growth at *t* = 0.) This type of nucleation, which is simply a special case of heterogeneous nucleation as discussed by Avrami [3], is for convenience termed *pseudohomogeneous* in this paper. So long as the rate of production of nuclei does not fall off too rapidly due to depletion of the flat surfaces, the behavior of such a system will in many important respects be experimentally similar to that of one where the nucleation is truly homogeneous. The similarity in the expressions describing the rate of heterogeneous nucleation of flat surfaces and true homogeneous nucleation has been noted on a number of occasions (see, for example, the remarks of Holloman [4]. However, pseudohomogeneous nucleation will be energetically favored over the corresponding homogeneous process, and the energy parameters that describe the rate of injection of primary nuclei in the pseudohomogeneous process will generally be considerably smaller than those for the corresponding truly homogeneous mechanism. Thus, the attainment of highly reproducible isotherms through strong superheating is in itself insufficient reason for the assumption that the subsequent nucleation is homogeneous, but this still does not detract from the fact that a study of a system exhibiting pseudohomogeneous nucleation may provide certain information concerning the expected behavior of the corresponding homophase system with homogeneous initiation. If the number of heterogeneities is sufficiently small, or the flat surfaces only difficultly wettable by the crystalline phase, true homogeneous nucleation will tend to predominate. Reasons will be given for believing that homogeneous initiation was probably achieved for carefully selected and strongly superheated specimens of poly (chlorotrifluoroethylene).

The second objective of the work involved an experimental study of the shape of the isotherms, particularly at high *x* values.

All of the isotherms could be quite accurately represented from very low up to moderately high *x* values by the useful phenomenological expression *x* = *x_w_* [1 −exp (−*Zt^n^/x_w_*)] attributable to Mandelkern [5], and Mandelkern, Quinn, and Flory [6]. In this region, *x_w_* and *n* were essentially constant, and the isotherms could therefore be superposed simply by rescaling the time. (The parameter *x_w_* is treated in this paper as a mean value of the *apparent* limiting degree of crystallinity in stage 1; note that *x_w_* causes the crystallization to proceed somewhat more slowly than the free bulk growth rate, *Zt^n^*.) However, at high *x* values, the isotherms rather abruptly entered a new regime where the degree of crystallinity changed extremely slowly with time, and where no constant value of *n* and *x_w_* could describe the shape of a given isotherm. Moreover, the various isotherms could no longer be superposed by rescaling the time. For convenience, the first part of an isotherm, where superposition holds, is called *stage 1*, and the later portion where it fails is termed *stage 2.* The stage 2 process is the principal hindrance to the isothermal attainment of a high degree of crystallinity in this polymer. A discussion is given concerning possible causes of the onset of stage 2. It is concluded that a high degree of impingement is the cause of this phenomenon.

The third objective of the work was to give a theoretical account of the rate constants that control the rate of nucleation and growth in the stage 1 portion of the crystallization. Special emphasis was attached to calculation of the temperature variation of these quantities, and to the estimation of the size of the nuclei.

In a system with primary nuclei being born at uniformly later and later dates and then growing in a one-dimensional manner, the overall (bulk) crystallization rate, as measured, by *Z*_2_ in the expression for the free growth rate, *x*′=*Z*_2_*t*^2^, is proportional to the rate of injection of nuclei *I*, the lineal growth rate of the rods *G*, and the area of the growing crystal face, $\pi {\bar{r}}^{2}$. Experimental values of *Z*_2_ were obtained at various temperatures by analysis of the *n* = 2 isotherms. Information concerning the temperature dependence of *G* was obtained by an analysis of the *n* = 1 seed-crystal isotherms, since under appropriate circumstances the overall rate of crystallization of a specimen where the nuclei are seed crystals present at *t* = 0 depends directly on the lineal growth rate. With the temperature variation of *Z*_2_ and *G* known experimentally, the temperature dependence of *I* was therefore determined also. Our task, then, was that of giving a theoretical discussion of *Z*_2_, *I*, and *G*.

The rate of injection of nuclei is described generally in terms of a cylindrical primary nucleus of radius *r* and length *l*, where *r* and *l* may be either variable or constant. This nucleus is endowed with interfacial free energies *σ_s_* and *σ_e_* on the side and ends respectively, and a residual edge free energy, *ϵ*. The residual edge free energy is shown to be unimportant in determining the rate of nucleation in the present case. In the theoretical treatment, the thermodynamic driving force, Δ*f*, is represented by a more accurate expression [7] than is commonly employed in similar calculations. The homogeneous nucleation rate in the region studied experimentally (170° to 200° C) is treated in terms of a specialized primary nucleus of fixed length *β* and a variable radius *r.* This model, which is applicable in a relatively extended temperature interval called “region B”, leads to a nucleation rate of the form $I_{B}\sim \exp(-\Delta F_{p}^{*}/RT)\exp(-\beta /T^{2}\Delta T)$, where *β* is a constant, $\Delta F_{p}^{*}$ the free energy of activation of the short-range diffusion process at the interface, *T* the absolute temperature, and Δ*T* the difference between the melting point and the crystallization temperature. It is demonstrated that this model does not imply that *σ_e_* is identical to zero. The model with both *r* and *l* variable is theoretically valid in region A, which exists close to the meltirg point, and leads to $I_{A}\sim \exp(-\Delta F_{p}^{*}/RT)\exp(-\alpha /T^{3}\Delta T^{2})$, where *α* is a constant. It is demonstrated conclusively that *I_B_*, and not *I_A_*, fits the data obtained between 170° and 200° C, showing that the nucleus with fixed *l*_0_ is the correct one to employ in this range. The temperature dependence of the lineal growth rate is described in terms of a secondary or growth nucleus of length λ_0_ that forms on the growing face (end) of the crystallite. The growth law is of the form $G\sim \exp(-\Delta F_{g}^{*}/RT)\exp(-\gamma /T^{2}\Delta T)$. The quantity *l*_0_ is numerically equal to λ_0_. The treatment of *G* is thus similar to that proposed in another connection by Burnett and McDevit [8]. The bulk crystallization rate constant, *Z*_2_, is shown to accurately conform to a relation of the form $Z_{2}\sim \exp[-(\Delta F_{p}^{*}+\Delta F_{g}^{*})/RT]\exp[-(\beta +\gamma )/T^{2}\Delta T\gamma )$., where *β* = *γ.* Estimates of the dimensions of the primary and secondary nuclei are given.

Although the principal aim of the present work was the elucidation of the bulk crystallization process, mention is made of certain aspects of spherulitic growth. A more detailed discussion of spherulitic growth, which to a certain extent supplements the work of Price [9] on poly(chlorotrifluoroethylene), will be given elsewhere [10]. It will be shown here that the *bulk* crystallization that takes place in this polymer subsequent to strong superheating is *not* of a three-dimensional or spherulitic character in the temperature range studied. It is essential to bear this point in mind while reading the paper. On theoretical grounds, it is shown to be reasonable to suppose that the intrinsic bulk crystallization process might involve three-dimensional growth close to the melting point, but this problem was not studied experimentally because of the extreme slowness of the crystallization at such temperatures.

## 2. Experimental Procedure

### 2.1. Materials

The specimens of poly(chlorotrifluoroethylene) that were used in this research were laboratory samples of Kel-F grade 300 polymer which were kindly supplied to us by H. S. Kaufman of the Minnesota Mining and Manufacturing Company. The material was supplied in the form of air-free molded sheets approximately 2 mm thick. The number average molecular weight was stated to be approximately 415,000.

It is especially important to note that it was necessary to select sheets that were as free as possible of heterogeneities. The basic criterion used in making the selection was the ability of a reasonable degree of superheating to yield highly reproducible bulk crystallization isotherms (in a subsequent crystallization) that were essentially independent of the superheating conditions. Conformity with this condition indicated that enough of the heterogeneities had been rendered inactive to permit the homogeneous or pseudohomogeneous process to make its appearance. Only a few of the sheets supplied met the stated requirements.

A spherulite count obtained under specified conditions proved to be a useful measure of the number of heterogenities. The samples used in the bulk crystallization studies contained only 10 to 30 spherulities per mm^3^ after the specimens were superheated to 305° C, and the spherulites grown anywhere between 180° and 192° C. Samples with a considerably larger number of spherulites did not meet the reproducibility requirements stated above.

A further point concerning the selection of material is of importance. Under certain conditions, poly (chlorotrifluoroethylene) is known to possess surface nucleation that causes the specimens to exhibit a grainy appearance when viewed normal to the surface with crossed nicol prisms [11]. Only those samples that were shown by sectioning studies to be sufficiently free of this phenomenon were used in the research.

Only relatively fresh specimens were employed, since these led to the most reproducible results. In the course of repeating some of the work, it was discovered that material stored over a year in air showed a tendency toward irreproducible behavior. Such aged specimens often yielded low *n* values. This may have been the result of excessive surface nucleation.

### 2.2. Volume Measurements and Crystallization Isotherms

The specific volume of the specimens was measured by weighing them in silicone oil, using the same apparatus employed in earlier studies on this polymer [2]. The general procedure used in making the runs was as follows. First, the polymer was suspended on a fine wire and heated in a stirred air bath to a preselected initial temperature, *T*_1_ for a time sufficient to bring the entire specimen to thermal equilibrium. This usually took approximately 10 min. The specimen was then plunged into a silicone oil bath operating at a predetermined crystallization temperature, *T*_2_. The sample was then weighed in the oil at times appropriate to the rate of crystallization, and the specific volume calculated for each reading.

The crystallization was assumed to begin when the specimen reached *T*_2_; this usually occurred 1.5 to 3 min after it was plunged into the oil bath. For a process with a strongly negative temperature coefficient, such as the one studied, this introduces only a small error, certainly not exceeding 1 min in the zero of time. In cases where the crystallization was quite rapid, as for *T*_2_<180° C, a small temperature correction was made for self-heating of the sample. The samples used in the investigation generally weighed from 1 to 5 g.

The very small amount (always less than 0.2 percent) of silicone oil sorbed by the specimens in the course of repeated experiments did not affect the rate of crystallization. The samples did not become discolored, and no bubbles or other evidence of degradation was ever observed in fresh specimens. We note in the latter connection that identical isotherms were obtained at a given growth temperature even after the sample had been heated many times to *T*_1_ = 305° C for the brief period indicated.

The isotherms at a given growth temperature are independent of the time the sample was held at *T*_1_ prior to crystallization.

The specific volume values were converted directly to the degree of crystallinity, *x*, which is defined as the *mass fraction* of the sample that is crystallized, using the equation

$$x=\frac{{\bar{V}}_{l}-{\bar{V}}_{s}}{{\bar{V}}_{l}-{\bar{V}}_{c}}.$$ (1)

In this expression, ${\bar{V}}_{l}$ is the specific volume of the pure supercooled liquid, ${\bar{V}}_{c}$ the specific volume of the pure crystal, and ${\bar{V}}_{s}$ the specific volume of the sample, all at the temperature of the run. The specific volume of the crystal at the required temperature was calculated from the equation

$${\bar{V}}_{c}=0.45563+0.8079\times 10^{-4}T+0.874\times 10^{-7}T^{2},$$ (2)

and the specific volume of the supercooled liquid was obtained using

$${\bar{V}}_{l}=0.47337+2.199\times 10^{-4}T+2.943\times 10^{-7}T^{2}.$$ (3)

In these expressions $\bar{V}$ is in cm^3^g^−1^ and *T* is in ° C. Equations (2) and (3) were derived using a rigorous analysis of $\bar{V}-T$ data [2]. With the experimental error in ${\bar{V}}_{s}$ considered, the crystallinity scale defined by eqs (1 to 3) yields *x* values to better than 0.01 at low *x*, and about 0.03 at *x* = 0.5, in the temperature range where the crystallization studies were carried out. Evidence that lends strong support to the crystallinity scale defined by eqs (1 to 3) will be brought out later in this paper.

## 3. Bulk Crystallization Isotherms: Geometry of Growth and Type of Nucleation

### 3.1. Experimental Conditions Leading to Homogeneous or Pseudohomogeneous Nucleation

Turnbull [1] has shown that if a substance contains thermally stable (and wettable) heterogeneities possessing pores or cavities on its surface, crystalline embryos can persist in these on an equilibrium basis well above the ordinary melting point. Such a body will act as a center of growth as soon as the material is supercooled. By sufficient superheating, the embryos in the cavities can be melted out, thus rendering them inactive as nucleation centers. Other things being equal, the embryos in the larger cavities melt out first. In cases where the cavities are small, and where the heterogeneity is strongly wetted by the crystalline phase, the crystalline matter may persist hundreds of degrees above the usual melting point. Reproducible isotherms independent of superheating conditions or previous thermal history should be obtained provided that all, or nearly all, of such embryos are destroyed. It is readily demonstrated that such a condition can be achieved with relatively clean samples of poly- (chlorotrifluoroethylene).

**Figure 1:**
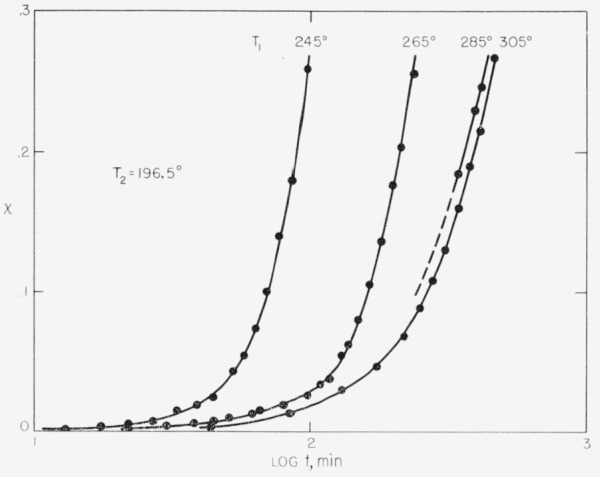
Effect of initial (superheating) temperature, T_1_, on isotherms obtained at a constant growth temperature, T_2_= 196.5° C. *x* is the mass fraction crystallized, and *t* is the time in minutes. Note the asymptotic approach to reproducible behavior with increasing *T*_1_.

The effect of various superheating temperatures, *T*_1_, on the isotherms for poly(chlorotrifluorethylene) obtained at *T*_2_ = 196.5° C is shown in figure 1. The equilibrium melting temperature, *T_m_*, for this polymer is close to 221° C [12], so that the degree of superheating ranges from 24° to 84° C. For low *T*_1_, the crystallization is noticeably dependent on *T*_1_. However, for high *T*_1_ the isotherms converge in a manner clearly suggesting that they are virtually independent of *T*_1_ in this region, and the rate of crystallization is slower.^2^ Results of a similar character were obtained with *T_2_* = 188.4° C and the same set of *T*_1_ shown in figure 1. Also a run carried out with *T*_1_ = 325° and *T*_2_= 196.5° C yielded an isotherm (not shown) that was identical within experimental error to that obtained with *T*_1_ = 305°, *T*_2_ = 196.5° C. These facts show that when the polymer is heated to the vicinity of 305° C prior to crystallization, the embryos in the crevices in the heterogeneites are virtually all destroyed. In such a situation, homogeneous or pseudohomogeneous nucleation initiates the crystallization process in the polymer.

### 3.2. Analysis of the Isotherms to Obtain *n*

**Figure 2:**
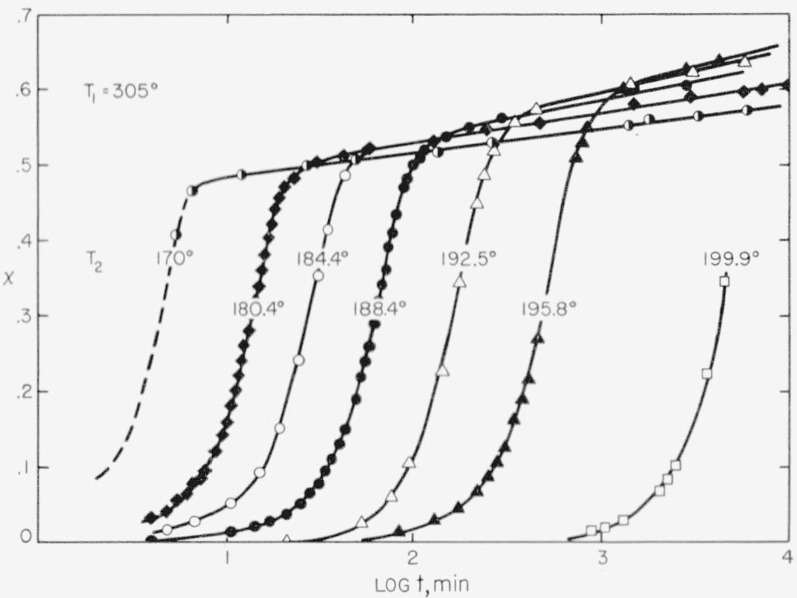
n=2 isotherms obtained at various growth temperatures, T_2_, for specimens subjected to strong superheating (T_1_ = 305° C) prior to crystallization. *x* is the mass fraction crystallized, and *t* is the time in minutes. The value *n* = 2 is a result of one-dimensional growth of nuclei born sporadically in time.

Plots of *x* versus log *t* obtained for various crystallization temperatures between 170.0° and 199.9° C are depicted in figure 2. All of these isotherms were obtained with polymer that was superheated to 305° C prior to the crystallization run, and are therefore those appropriate to homogeneous or pseudohomogeneous initiation. The data for the first part of the 170° run are somewhat unreliable owing to the rapidity of the crystallization. The bulk crystallization process has a strongly negative temperature coefficient in the temperature interval studied: the isotherms at low *T*_2_ arrive at a given (low or moderate) degree of crystallinity much sooner than those run at high *T*_2_. The existence of this strongly negative temperature coefficient shows that the bulk crystallization process is controlled by one or more nucleation mechanisms in the temperature range under investigation.

The two distinct stages of the crystallization are apparent in figure 2. In stage 1, the rate of crystallization at a given temperature is increasingly rapid. This stage strongly reflects the free growth rate of the crystals, and the analysis of *n* will be based on this portion of the isotherms. Stage 2 appears with only a slight premonitory effect, and is characterized by an extremely slow and nearly linear increase in the degree of crystallinity on the logarithmic time scale used. Stage 2 crystallization will be discussed in more detail in section 4.

The value of *n* in the expression for the free growth rate, *x*′=*Zt^n^*, will now be determined by an analysis of the isotherms. The free growth rate is the rate at which the polymer would crystallize if the nucleation and subsequent growth of each crystallite were independent of similar processes in other crystallites, a condition most apt to be fulfilled in the very early stages of the crystallization. Interest in the free growth rate is occassioned by the fact that *Z* is a quantity that can be calculated theoretically by a consideration of the elementary nucleation and growth mechanisms, and by the fact that *n* is intimately related to the type of nucleation and the geometry of growth.

The phenomenological expression [5,6]^3^

$$x=x_{w}\left[1-e^{-(Zt^{n}/x_{w})}\right]$$ (4)

is used as a starting point in analysis to obtain *n.* A derivation of eq (4) is given in appendix 9.1 to indicate the specialized meaning we have placed on *X_w_*, which is defined as a retardation parameter that reflects the mean value of the *apparent* limiting degree of crystallinity in stage 1. As shown in appendix 9.2, it is readily deduced from eq (4) that

$$n=(t/x)(\mathrm{dx}/dt)[1+(1/2)(x/x_{w})+\ldots ].$$ (5)

In this expression, *t* is the time as measured from the inception of the experiment, and *x* the degree of crystallinity at that time. Values of *n* at various degrees of crystallization may be obtained from the experimental values of *t, x*, and *d_x_/dt*, if a value of *x_w_* is assumed. Fortunately, *n* is quite insensitive to the choice of *x_w_* and it is sufficient for the purpose at hand to calculate *n* with *x_w_*= 1 and *x_w_*= 1/2. These values may be accepted as reasonable trial values for this parameter in the present case. Values of *x* up to 0.20 were used in the determination of *n.* This it was set in order to insure that each isotherm was well within stage 1, and to be certain that higher terms in eq (5) were unimportant. It was found that this method was more satisfactory than some of the curve-matching schemes sometimes used to estimate *n*, since a definite numerical value is obtained.

Values of *n* obtained in the manner described are given in table 1. It is evident from the results that *n* = 2.0 under the conditions of the experiments, which are those corresponding to homogeneous or pseudohomogeneous nucleation. (Note that the isotherm obtained with *T*_1_ = 285° C is also consistent with *n*=2.) It is extremely doubtful that the value of *n* lies outside the range 1.8 to 2.2. As indicated in the next to the last column in table 1, an isotherm calculated using eq (4) with *n*=2 and *x_w_*= 1 fits the observed data reasonably well up to a % value in the vicinity of 0.45. Lower values of *x_w_* usually give a poorer fit, indicating that *x_w_* ~ 1 in this case. The error in *x_w_* is undoubtedly rather large, the figures given being reliable to not much more than about 20 percent. Nevertheless, there is little doubt that eq (4) is a suitable method of representing the isotherms in the region indicated. At higher values of *x*, each experimental isotherm enters stage 2, and deviates markedly from that calculated from the phenomenological equation. The best values of the quantities *n* and *x_w_* are shown as bold face numbers in table 1.

The pseudoequilibrium degree of crystallinity, *x_m_*, for each *T*_2_ value is also shown in table 1. *x_m_* is defined as the degree of crystallinity at the onset of stage 2. The method of estimating *x_m_* is apparent from figure 2. The increase of *x_m_* with increasing *T*_2_ is definitely real, and is not an artificial result arising from an error in the crystallinity scale. (It is worth mentioning that this point is clearly apparent in the $\bar{V}-T$ diagram to be given in section 4.)

An approximate theoretical justification for using trial *x_w_* values in the range 1/2 to 1 for a system where *x_m_*~ 1/2 will be found in appendix 9.1.

**Figure 3:**
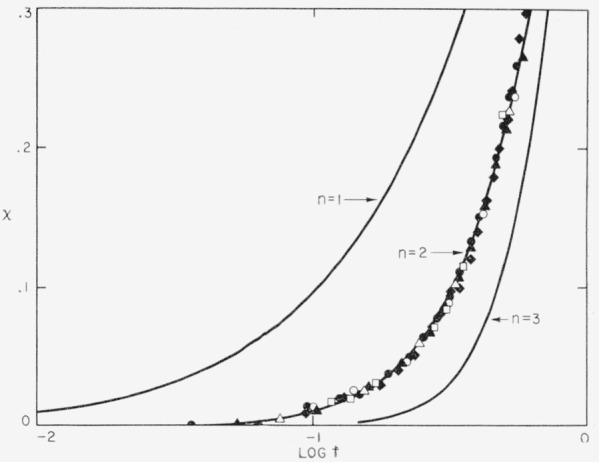
Superposition of experimental isotherms shown in figure 2 on isotherm calculated with eq (4) for case n=2. Isotherms calculated with eq (4) for *n* = 1 and *n* = 3 are shown for comparison. The symbols used to denote the experimental points for the various isotherms are the same as those used in figure 2.

The isotherms shown in figure 2 may be superposed by shifting them along the log *t* axis. A plot of such a superposition of isotherms is shown in figure 3. The points used in this plot are the same ones shown in figure 1, and these have been superposed on a phenomenological isotherm computed from eq (4) for the case *n*=2, and *x_w_*=1. The superposition is, of course, in accord with the mathematical properties of eq (4), and is equivalent to the superposition obtained using certain variables other than *x* [5] that measure the course of the crystallization. The superposition is excellent up to *x* = 0.3, and fair up to *x* = 0.4 to *x*=0.5 (see next to last column in table 1).

If it is assumed that the value of *n*=2 corresponds to homogeneous or pseudohomogeneous nucleation, i.e., to the case where the growing particles are born at later and later dates, then it follows that the geometry of growth is of a one-dimensional character. (It is shown in appendix 9.4. that *x′*=*Z*_2_*t*^2^, i.e., *n*=2, for the case of one-dimensional growth of objects born at later and later dates.) Any such conclusion concerning the geometry of growth rests squarely on the credibility of the arguments given earlier for the belief that strong superheating should lead to primary nuclei that are born at later and later times. At this point, therefore, it is considered that it is highly probable, rather than certain, that the geometry of growth in this polymer is one dimensional. What is clearly needed in an independent proof of the geometry of growth. This will be given in section 3.3. In order to facilitate this proof, it is necessary to indicate the conceivable alternative explanations for the *n*=2 isotherms, and to mention the unique character of certain other *n* values.

An *n*=2 isotherm can arise in two fundamentally distinct ways. It can be due to the one-dimensional growth of objects born at later and later dates, or to the two-dimensional (radial) growth of disk-like objects born at the same time. (These two cases are considered in appendix 9.3 and 9.4). This statement is subject to the provision that the growth process must be lineal, so that a given amount of material is deposited on a unit area of growing crystal face in unit time. It may safely be assumed in the case of poly(chlorotrifluoroethylene) that this holds true, at least in the first stage of the crystallization. Nonlineal growth in the relatively early stages of a crystallization is to be anticipated only in systems where long-range diffusion is required to bring the crystallizing species to the surface of the crystal. Thus, only the case of one-dimensional growth of objects born at later and later dates, and twodimensional growth of objects born at the same time need be considered as possible causes of the observed *n* = 2 isotherms. An independent proof that the geometry of growth is indeed one-dimensional in this polymer would therefore strongly reinforce the belief that the *n* = 2 isotherms are a result of objects born at later and later dates.

It is worth remarking that a similar problem exists for an *n* = 3 isotherm. Such an isotherm could be interpreted as being a result of threedimensional growth of objects born at the same time, or two-dimensional growth of disk-like objects born at later and later dates. Fortunately, however, *n* = 1 and *n* = 4 isotherms have unique interpretations, subject of course, to the provision that the growth be lineal. Thus, an *n* = 4 isotherm can arise only for the three-dimensional (spherical) growth of objects born sporadically in time, and an *n* = 1 isotherm can arise only for one-dimensional growth of objects born at the same time.^4^ The latter fact, when used to interpret the experiments with seed crystals to be described in the following section, will provide the required proof that the geometry of growth in poly(chlorotrifluoroethylene) is indeed one-dimensional. This will also resolve any uncertainty in the suggested interpretation of the *n* = 2 isotherms.

### 3.3. Nucleation With Seed Crystals

If the crystallites formed in the *n* = 2 crystallization are actually a result of one-dimensional growth of nuclei born at later and later times as has been postulated, it is clear that if seeds derived from these crystals are caused to exist in a sample of polymer, and these seed nuclei used to initiate a crystallization at *t* = 0, the resultant isotherm should conform, at least in its early stages, to eq (4) with *n*≅1. (It is shown an appendix 9.3 that the free growth rate for such a system is *x*′ = **Z**_1_*t*.)

The preparation of a sample containing the appropriate type of seed crystals is easily accomplished. First, the specimen is crystallized at a certain growth temperature, such as 188° C, after first superheating it to 305° C. The corresponding isotherm is of the *n* = 2 type. This process produces vast numbers of tiny crystallites in the specimen. The sample is then heated to a temperature *T*_1_ that is a little *below T_m_* but close to the quasi-equilibrium melting point, *T_m_*′, in order to melt out all but a very small fraction of the crystallites.^5^ The equilibrium melting temperature of poly(chlorotrifluoroethylene) is 221° C, and the quasi-equilibrium melting point for a sample crystallized at 188° C is about 216° C [12]. The remaining crystallites are the largest in the specimen, the smaller ones having melted out, and comprise the requisite type of seed crystals. Though numerous seed crystals are present, the specific volume of such a sample is experimentally indistinguishable from that of the pure supercooled liquid at the same temperature.

The seed-crystal isotherm is obtained by dropping the temperature of the “seeded” sample to a convenient crystallization temperature, *T*_2_, and making the run in the usual manner. The seed crystals are, of course, all active as “heterogeneous” nucleation centers at *t* = 0. Isotherms obtained in this way were analyzed to obtain *n* as was described in the previous section. The results of three of the runs are given in table 2. It is seen that the value *n* = 1.0 provides the best description of the data. *The value n* = 1 *can only be interpreted to mean that the crystallization in the seeded specimens was due to one-dimensional growth of objects born at the same time. Further*, *this finding provides the strongest kind of confirmation for the supposition made earlier that the n* = 2 *isotherms were a result of one-dimensional growth of objects being born at later and later dates, i.e., by homogeneous or pseudohomogeneous nucleation.*

The specimens in the *n* = 1 seed-crystal runs attained a given degree of crystallinity considerably faster than the samples in an *n* = 2 run at the same growth temperature, despite the acceleration of the crystallization resulting from the continuous injection of centers in the latter case. This is a result, of course, of the fact that the numerous seed crystals all start growing at *t* = 0 in the *n* = 1 runs. The *n* = 1 seed-crystal runs were carried out at the relatively high *T*_2_ values indicated in order to allow the rate of crystallization to be measured easily.

The result *n* = 1 is definitely not caused by surface nucleation in these experiments. Microscopic observation of sectioned specimens showed that the crystallization did not originate at the surfaces; the scattering of light due to the crystals formed clearly indicated that they existed throughout the body of the specimen. Thus the *n* = 1 isotherms were a result of a bulk crystallization effect. It is also perfectly clear from the conditions of the experiment that the result *n* = 1 is not caused by continuous homogeneous injection of nuclei that do not grow.^4^

**Figure 4:**
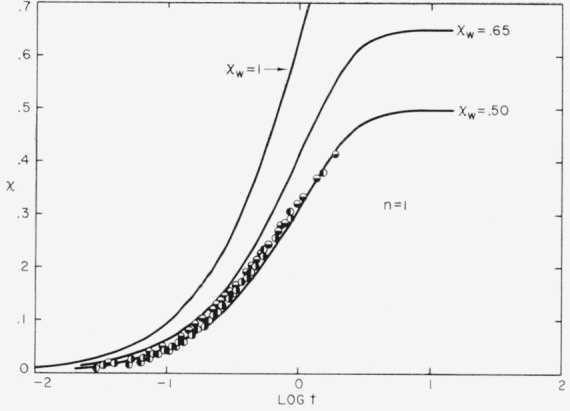
Superposition of the experimental n = 1 isotherms obtained by initiation with seed crystals on isotherms calculated using eq (4) with n = 1 and various values of x_w_. ◑ *T*_1_ = 216.2°, *T*_2_ = 200° C; ◒ *T*_1_ = 215.6°, *T*_2_ = 196.2° C; ◐ *T*_1_ = 215.6°, *T*_2_ = 200° C. The value *n* = 1 is a result of one-dimensional growth of pre-determined nuclei (seeds) present at *t* = 0.

A superposition-type plot of the *n* = 1 isotherms is shown in figure 4. The isotherms shown are those mentioned in table 2. Curves calculated using eq (4) with *n* = 1 and various values of *x_w_* are shown as solid lines. It is seen that *x_w_* ≅ 0.55 provides a good fit of the data from *x* = 0 up to *x* = 0.3 to 0.4. The best values of *n* and *x_w_* for this type of isotherm are indicated as bold face numbers in table 2. The mean experimental value of *x_w_* obtained for the *n* = 1 isotherms is correct to within about 10 percent.

A *x_w_* value that diminished somewhat as the degree of crystallinity increased would definitely fit the data slightly better. This point will prove to be of significance in an ensuing discussion on the nature of impingements.

## 4. Isotherms at High *x*: Stage 2 Crystallization

The objective of this section is to bring out certain points concerning the shape of an observed isotherm, especially at a high degree of crystallinity where the inception of stage 2 introduces strong retardations to the crystallization process. The nature of stage 2 is of special interest when considered in the light of the difficulties encountered in attaining very high degrees of crystallization in linear polymers.

**Figure 5:**
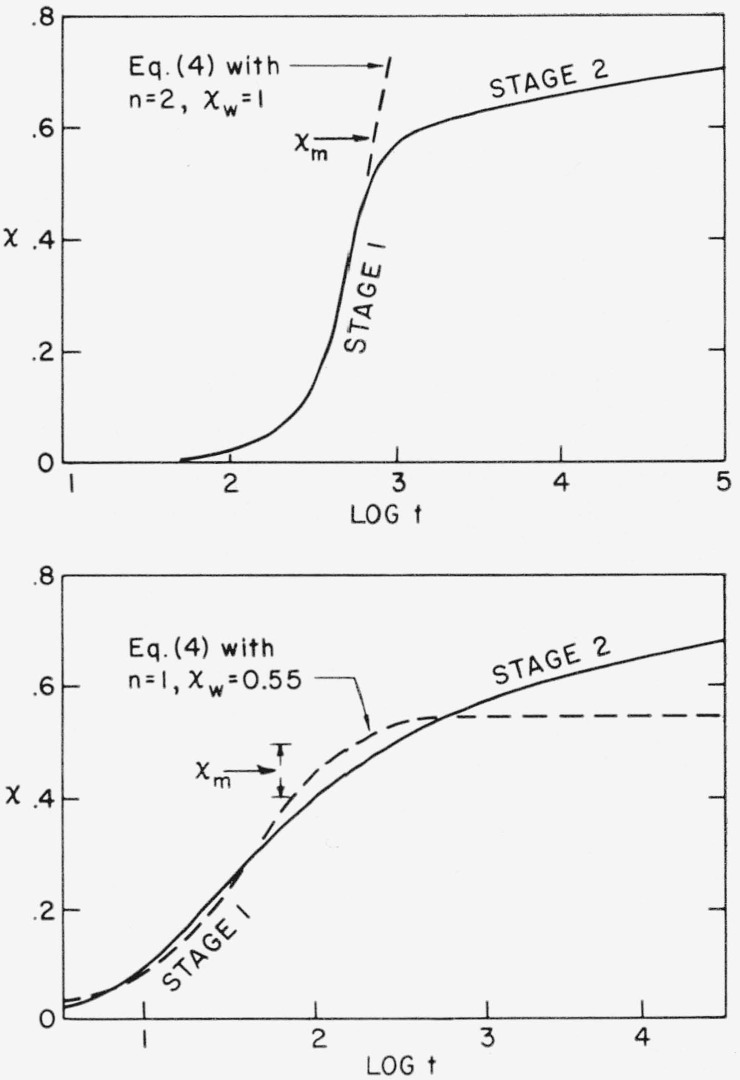
Diagram showing transition from stage 1 to stage 2, and the extent to which eq (4) (dashed lines) fits the experimental isotherms (solid lines) for the n = 1 and n = 2 cases. The *n* = l curve shown was obtained with *T*_1_ = 218.0°, *T*_2_ = 196° C, and the *n* = 2 curve was obtained with *T*_1_ = 305°, *T*_2_ = 196° C. *x* is the mass fraction transformed and *t* is the time in minutes. The symbol *x_m_* represents the approximate value of the pseudoequilibrium degree of crystallinity.

Consider first the *n* = 2 isotherms shown in figure 2. The retardations to the crystallization in stage 1 of these isotherms are comparatively weak, and are adequately described up to moderately high *x* values by the parameter *x_w_~*1 in eq (4). The stage 1 portion is superposable as shown in figure 3. The situation is entirely different after the onset of stage 2 at *x* ≅ 0.4 to 0.6. The crystallization becomes extremely slow, indicating the interdiction of a very strong retardation, and no fixed values of *Z*_2_, *n*, and *x_w_* with eq (4) are capable of reproducing any significant portion of the stage 2 portion of the isotherm. The stage 2 portions of the isotherms are not superposable by rescaling either the time or the degree of crystallinity. Equation (4) with *n* = 2 and *x_w_* = 1 leads to an isotherm which rises on past the onset of stage 2, as shown in figure 5 (upper diagram).

It is emphasized that the value *x_w_*~1 found experimentally for the *n* = 2 isotherms is not particularly accurate, and is probably somewhat high. A small and otherwise negligible *n* = 3 component due to a remnant of heterogeneously induced spherulitic growth is suspected to be present in the *n* = 2 runs, and this would cause *x_w_* to assume somewhat high values. Also, slightly conical growth would lead to high *x_w_* values. Thus, the fact that *x_w_* is considerably greater than the pseudoequilibrium degree of crystallinity, *x_m_*, in the *n* = 2 case may be partly an artificial result, and the true value of *x_w_* may well be closer to *x_m_* than is indicated by most of the data. However, none of the above detracts from the fact that eq (4) provides a good representation of the data in stage 1, and the fact that the retardations in stage 1 are much weaker than those in stage 2.

The *n* = 1 isotherms also exhibit typical stage 2 crystallization. In the case of the *n* = 1 isotherms, the onset of stage 2 is rather diffuse, but evidently becomes important at around *x* = 0.4 to 0.5. The general situation for the *n* = 1 isotherm is depicted in figure 5, (lower diagram). The properties of the stage 2 part of the isotherm at high *x* in the *n* = 1 case are very similar to those described above for the *n* = 2 case. The value *x_w_*≅0.55 that describes the retardations in stage 1 of the *n* = 1 isotherms is attributable principally to the effect of impingements and entanglements (see below).

A slow crystallization process evidently similar in character to the stage 2 crystallization investigated here has previously been noted in crystallizable polymers by Collins [13], Kovacs [14], and others.

The experiments clearly indicate that the parameter *x_w_* in eq (4) is not generally to be identified with the true equilibrium degree of crystallinity. To show this, we need only consider the *n* = 1 type isotherm, for which the relatively reliable value *x_w_*≅ 0.55 obtains. It has been demonstrated that poly(chlorotrifluoroethylene) can readily be crystallized up to *x* = 0.82 [2], and even this figure is certainly not the highest attainable. Thus *x_w_* is clearly less than the equilibrium degree of crystallinity in this case. Another point is that in the experimental *n* = 1 isotherm shown in figure 5 (lower diagram), *x* is seen to continue well past the value *x*≅0.55, again demonstrating that *x_w_* is not the equilibrium degree of crystallinity. The fact that *x_w_* is roughly unity for the *n* = 2 isotherms does not in our view imply that *x_w_* is to be identified with the equilibrium degree of crystallinity in these cases, especially in view of the uncertainty in the experimental value of *x_w_* for such isotherms.

The experimental results suggest that the retardation parameter *x_w_* is not to be generally taken as being identical to the pseudoequilibrium degree of crystallinity, *x_m_*, though, this point is difficult to establish with certainty. The most clear-cut experimental evidence is obtained from the *n* = 1 isotherms where the value *x_w_*≅0.55 is established within narrow limits. Our best estimate of *x_m_* in this case yields *x_m_* ≅ 0.45 (see table 2). This numerical estimate of *x_m_* is somewhat doubtful, but our analysis nevertheless renders it highly likely that *x_m_* is somewhat less than The situation with the *n* = 2 isotherms is that *x_m_* can be determined quite accurately (see fig. 5, upper diagram), but that *x_w_* is more uncertain. Nevertheless, it would appear that *x_m_* is less than *x_w_* in this case. At the very least, the experimental results for the *n* = 1 and *n* = 2 isotherms can hardly be interpreted to mean that *x_w_* is precisely equal to *x_m_*.

The experimental situation concerning the onset of stage 2 in the *n* = 1 and *n* = 2 cases can be summarized as follows. In the *n* = 2 case, the stage 2 process intervenes rather abruptly at *x_m_*, where *x_m_* is 0.4 to 0.6 depending on the crystallization temperature, and prevents the experimental isotherm from pursuing its original *“n* = 2, *x_w_*~I” course. However, the *x_w_* value quoted is undoubtedly somewhat high as a result of certain extraneous effects. In the *n* = 1 case, the experimental isotherm essentially completes its *“n* = 1, *x_w_*≅0.55” course near *x_m_* = 0.45, and the stage 2 process supervenes. In both cases, it is the stage 2 mechanism (and the limited patience of the investigator) that hinders the isothermal attainment of high degrees of crystallinity.

The above remarks serve to show part of the experimental basis for referring to *x_w_* ns a “retardation parameter” or as the “apparent limiting degree of crystallinity in stage 1,” rather than assuming it is generally equivalent to the pseudoequilibrium degree of crystallinity. A theoretical justification for this type of definition will be mentioned shortly (see also appendix 9.1). The theoretical analysis will bring out the fact that *x_w_* will depend to a certain extent on *x*, and that *x_w_* may often be fairly close to *x_m_*.

Some possible causes of the onset of stage 2 type crystallization will now be considered. It is of interest first to point out certain apparently attractive hypotheses that cannot explain the observed results.

First, the onset of stage 2 in the *n* = 2 isotherms cannot be due to a rapid depletion of flat surfaces on heterogeneities that can act as sites for pseudohomogeneous nucleation. This is demonstrated by the fact that stage 2 of an *n* = 2 isotherm is quite similar to that found for the *n* = 1 seed crystal isotherms at high *x* (fig. 5). In as much as the seed crystals all lead to nuclei born at *t* = 0 in the *n* = 1 case, so that depletion of nuclei cannot be the cause of the onset of stage 2 in such an isotherm, it follows that site-exhaustion effects on heterogeneities cannot be the cause the onset of stage 2 in the *n* = 2 isotherms. The second hypothesis would be that the degree of crystallinity at the onset of stage 2 was the equilibrium degree of crystallinity, but this is disproved by the observation that *x_m_* is always well below the maximum degree of crystallinity attained. It can thus be stated with certainty that *x_m_* is not to be explained in terms of the well-known equilibrium statistical-thermodynamical theory of crystallization due to Flory [15], where the limitation on the degree of crystallinity is basically a result of the exclusion of chain ends from the crystal. The name “pseudoequilibrium degree of crystallinity” is thus aptly applied to *x_m_*.

The probable explanation of the onset of stage 2 is found in the work of Lauritzen [16], who has analysed theoretically the retardations in a system of rods or disks that grow normal to the radius in terms of impingements. Lauritzen has rigorously solved the following problem relevant to the present work. Let *n*_0_ nuclei be present per unit volume at *t* = 0, and assume these to be at random positions in space. Permit these nuclei, which all have radius $\bar{r}$, to grow at a constant rate in a one-dimensional manner at random orientations until they impinge on another growing particle, and calculate the fraction crystallized as a function of time. This calculation should correspond reasonably closely to the *n* = 1 seed crystal case studied experimentally in this paper.

The results of Lauritzen’s calculations will be couched in terms of the behavior of *x_w_* in eq (4) with increasing *x.* This will serve not only to provide a certain theoretical justification for the use of the phenomenological relation, but will also bring out more clearly the true nature of the retardations, and the limitations of eq (4).

The results of Lauritzen’s calculations may be summarized as follows. The theoretical isotherm starts out in a manner quite similar to that calculated by eq (4) with a *x_w_* value that is well below unity. This initial value of *x_w_* is denoted *x_w(i)_*, and has a theoretical value of 0.43. In our view, this provides a theoretical justification for the use of eq (4) at relatively low *x* values, with *x_w_* being considered as a retardation parameter. As the degree of crystallinity increases, *x_w_* tends to drop somewhat, but the retardation is still weak enough so that the crystallization may be regarded as being of an essentially superposable type. The crystallization finally comes to a virtual stop due to a massive number of impingements at a degree of crystallinity well below unity that may be identified with *x_m_* The value of *x_w_* at the end of the impingement process is *x_m_.* The value of *x_m_* calculated on the impingement model depends on the scaling parameter $n_{0}{\bar{r}}^{3}$; a large value of this parameter leads to a high *x_m_*, and a small value leads to a low *x_m_.* It is emphasized that these results refer to *n* = 1 class isotherms.

The theoretical calculations clearly demonstrate that *x_w_* in eq (4) will vary somewhat with *x*, and is therefore best considered as a retardation parameter in the relatively early stages of the crystallization, rather than the pseudoequilibrium degree of crystallinity, a value it approaches only toward the latter part of stage 1. The experimental values quoted for *x_w_* in table 2 should therefore be considered as average values. The same is undoubtedly true of the *x_w_* values for the *n* = 2 isotherms in table 1. It is reasonable on theoretical grounds to expect *x_w_* to be rather close to *x_m_* in certain instances; this will especially tend to be the case where $n_{0}{\bar{r}}^{3}$ is such that *x_m_*≅*x_w(i)_.* The reader is referred to appendix 9.1 for further details concerning impingement theory.

The general type of behavior expected from the theoretical calculations is what is found for the experimental *n* = 1 isotherms, where *x_w_*≅0.55. As mentioned in section 3.3, *x_w_* falls slightly with increasing *x.* A rough estimate would be that *x_w(i)_* ≅ 0.65 and *x_m_* ≅ 0.45. The value of *x_w(i)_* is higher than the theoretical value of 0.43, but this is probably mostly a result of the fact that all impingements are not effective in stopping growth as was assumed in the theory (see below).

The impingement theory for rod-like objects born at later and later dates is mathematically formidable, and has not been solved rigorously except for very low *x*, but there is good reason to believe that it will lead to a virtual cessation of the crystallization at moderate *x* values due to a massive degree of impingement. The value of *x_w(i)_* in this type of system can, however, be estimated with some accuracy [16].

The impingement theory described above is based on the idea that a crystal stops growing when it touches another crystal. In many situations, such as when one crystal runs into another at essentially a right angle, this seems sufficiently realistic. In the case of certain types of “grazing” collisions the assumptions used may be too stringent, with the result that the predicted values of *x_w(i)_* and *x_m_* would be somewhat low. Nevertheless, impingement theory provides a convincing physical explanation for the behavior of the retardation parameter *x_w_*, and the origin of the pseudoequilibrium degree of crystallinity *x_m_*.

It is emphasized that the impingement model treated by Lauritzen is of a general enough nature to represent approximately a number of physically conceivable situations that could cause a crystallite to stop growing, at least at a normal pace, far short of its “equilibrium” length. For example, some of the crystallites may actually stop growing one- dimensionally because of chain entanglements arising from situations where the same polymer molecule becomes involved in more than one growing crystallite. Such an entanglement might be considered as an impingement. In any event, it would appear for poly(chlorotrifluoroethylene) that the mean crystallite length at the onset of stage 2, i.e., at *x_m_*, is largely determined by kinetic factors having to do with how rapidly the rate of nucleation and growth causes the crystallites to suffer numerous impingements. It will emerge later that the mean radius is affected by similar considerations. The role of kinetics in influencing the dimensions of the crystallites in polymers crystallized by procedures of ordinary duration cannot be overlooked.

It has been observed that the quasi-equilibrium melting point of the polymer, *T'_m_*, increases slightly as stage 2 progresses. An increase of melting point on prolonged storage has been noted for certain crystallizable polymers on a number of previous occasions, and has been employed as a basis for estimating *T_m_* [5]. The increase of *T'_m_* noted as stage 2 progresses may be ascribed to either a gradual increase in radius or length of the rods, or both. Such increases could be a result of relaxation of impingements. Both types of process would tend to establish larger crystals more consistent with true equilibrium conditions. The gradual increase of crystallinity with time in stage 2 is probably mainly the result of such effects, and in addition, there may be a gradual injection of nuclei into the amorphous interstices between the impinged crystallites.

There is an indication in figure 2 that the stage 2 process is more rapid the higher the temperature. It would thus appear that some slow diffusion mechanism was the rate-determining step in the stage 2 process.

In subsequent sections of this paper, the primary nucleation mechanism in a bulk polymer will be treated as taking place by a lateral accretion of segments belonging to various polymer molecules to form a bundle-like nucleus. This nucleus is then assumed to grow both radially and lengthwise at varying rates. We refer to such growth as “kinetic” growth, and to the resultant crystallite as a “kinetic” one. The object of the discussion immediately following is to bring out the fact that the kinetic growth of such a primary nucleus can hardly evade encountering some chain ends, with the result that the corresponding kinetic crystal may contain some such objects, and thus be slightly less stable and less dense than the true equilibrium one.

In the kinetic picture of the growth of a polymer crystal from a bundle-like primary nucleus, chain ends are certain to appear on the growing (end) face of the crystal. The probability of such an event will depend on the molecular weight. If these chain ends are large in both number and size, the one-dimensional growth may be seriously disrupted, at least locally. If on the other hand, the chain ends are small, e.g. a −*CF*_3_ group, and their number not too great, then a somewhat disordered crystal containing some chain ends may form initially. Other things being equal, such a crystal would have a slightly higher free energy than one containing no chain ends. In a high molecular weight material containing small chain ends, the lineal growth process should not be seriously disrupted by the occasional inclusion of chain ends in the “kinetic” growth of the crystal. It should be pointed out that the equilibrium polymer crystal will contain very few and perhaps no such chain ends. (Flory [15] has treated the equilibrium case with no chain ends in the crystal in detail.) The point here is that if the “kinetic” crystal does contain some small chain ends—and we consider this probable in many cases—the crystal will tend to seek its minimum free energy at a given temperature by allowing the chain ends to diffuse to the surface or end of the polymer crystal. This would be an exceedingly slow process that had a positive temperature coefficient. This may be one of the mechanisms involved in stage 2. The slight increase of *T'_m_* on prolonged storage noted in stage 2 may thus be partly a result of the increased perfection of the crystals, and a small part of the increase of density may be due to the same effect. The mechanism mentioned would lead to a relief of internal strain in the polymer. However, it is probable that the main cause of the increase if *T'_m_* on storage is the slow growth of crystallite size resulting from the relaxation of impingements and entanglements mentioned above.

The melting point of a sample that has been crystallized part way into stage 2 at *T*_2_, where *T*_2_ is below *T_m_*, is not only low but broad as well. Samples of this type correspond closely to the moderately crystalline specimens commonly encountered in practice. In such material, the broad melting curve is certainly principally a result of the fairly wide distribution in the size of the crystallites in the system. A likely source of this distribution would appear to be fluctuations of the radius about the mean value ¯, and similar fluctuations about the mean length $\bar{l}$, that result from impingement of the growing crystallites on one another. The shape of the broad melting curve observed for this type of specimen is not to be interpreted in terms of the equilibrium theory of the melting of crystalline homopolymers. Any attempt at the precise application of such a theory should be reserved for polymer that has progressed considerably further into stage 2, i.e., much closer to an equilibrium condition.

None of the above in any way contradicts or refutes the concept that a crystalline polymer possesses an equilibrium degree of crystallinity or an equilibrium melting temperature, the latter being defined as the melting point of the largest and most perfect unstrained crystal attainable [5]. However, it does illuminate some of the factors that impede the attainment of an equilibrium degree of crystallinity, and the measurement of the shape of the equilibrium melting curve below *T_m_*.

**Figure 6:**
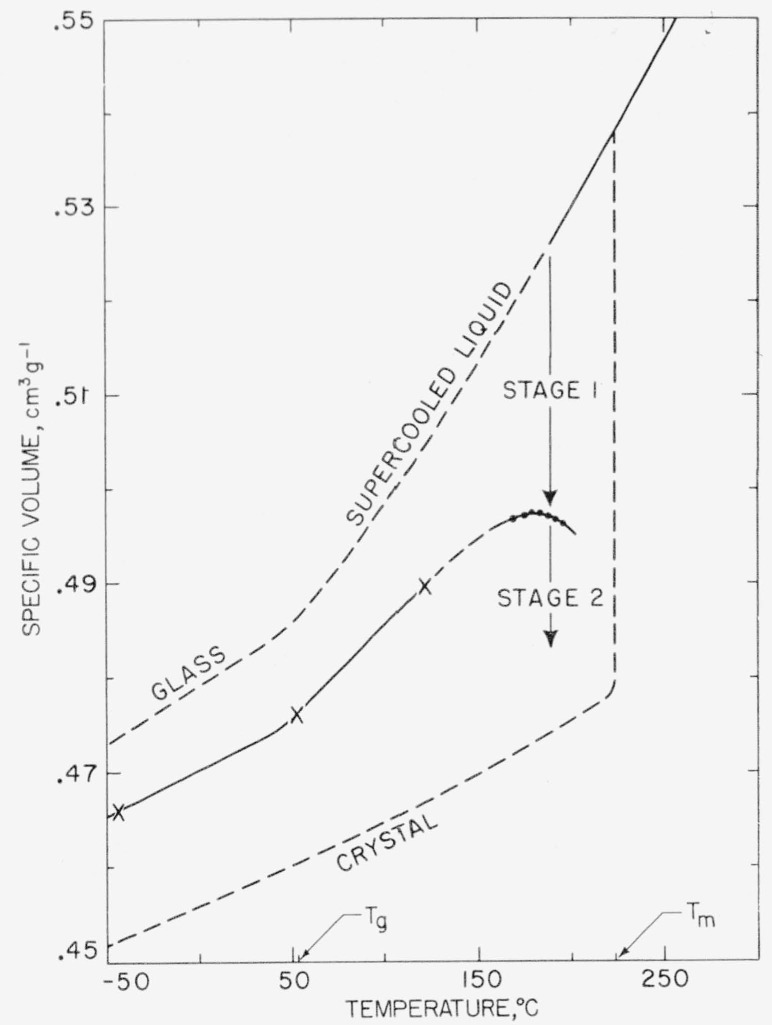
Specific-Volume—Temperature diagram showing the locus of the transition between stage 1 and stage 2. The line ӿ-ӿ-ӿ indicates the specific volume obtained for a “quenched” specimen ~2 mm thick. Extremely rapid quenching of sufficiently thin films yields material with a specific volume that is much closer to the supercooled liquid or glassy state curve.

It is of interest to note where the onset of stage 2 takes place with respect to a $\bar{V}-T$ plot for poly (chlorotrifluoroethylene). The approximate demarcation line between stage 1 and stage 2 for the *n* = 2 isotherms is indicated in figure 6. The demarcation was obtained by drawing a straight line through the main part of stage 1 and stage 2 portions of each isotherm plotted as $\bar{V}$ versus log *t*, and noting the point of intersection. On a degree of crystallinity scale, such an intersection corresponds to *x_m_.* The $\bar{V}-T$data for the pure supercooled liquid, glassy, liquid, and crystalline states shown in the diagram are those obtained in a previous study [2].

Two interesting points are evident in figure 6. First, stage 1 accounts for a larger amount of the volume change (and percentage crystallization) near *T_m_* than it does at somewhat lower temperatures. (This means that *x_m_* tends to increase with increasing *T*_2_, as may be seen in table 1 and figure 2). The second point involves the nature of “quenched” samples. A specific volume curve for a “quenched” sample ~2 mm thick taken from earlier work [2] is denoted in the diagram by the line ӿӿӿ. This is seen to be simply a continuation of the junction between stage 1 and stage 2. Such a “quenched” specimen is one that has traversed most of stage 1, but little if any of stage 2, thereby achieving the appropriate pseudoequilibrium degree of crystallinity while it was being rapidly cooled. Hence, the extreme sluggishness of the stage 2 mechanism, together with its rather abrupt onset, accounts for the strong similarity in the specific volume data at room temperature obtained for “quenched” sheets of this polymer even though they ranged from 1 to 3 mm thick and were subjected to quenching procedures of varying efficiency [2, 17]. Extremely rapid quenching, such as is possible with very thin sheets, can cause the material to become practically completely amorphous (see section 6.4).

Stage 2 isotherms similar to the ones shown in figure 2 can be obtained for poly(chlorotrifluoroethylene) by first quenching specimens ~2 mm thick from 305° to 0° C, reheating to the appropriate *T*_2_ value, and then making the run in the usual way. The quenching step carries the polymer through stage 1.

The stage 2 mechanism should not be confused with the slow and small decrease of specific volume at constant temperature that can take place in completely amorphous materials in the immediate vicinity of the glass transformation temperature, *T_g_*, even if a similar “logarithmic” law is followed in the two cases. The small decrease of specific volume with time in amorphous materials near the nominal value of *T_g_* is basically a result of the fact that *T_g_*, as ordinarily dealt with, depends on the rate of measurement. Such isothermal volume changes are not found in the supercooled liquid state of completely amorphous systems if the temperature is much above or below *T_g_.* The stage 2 mechanism in poly(chlorotrifluoroethylene) is principally due to an increase in the degree of crystallinity, and not to some “compacting” of the segments that takes place with the passage of time solely in the supercooled liquid state. This is shown by two facts: (a) the volume change in stage 2 is much too large to be reasonably accounted for by a change in the supercooled liquid state (in some cases the stage 2 process carries the crystallization from *x* ≅ 0.4 to *x*≥0.80) and (b) the volume change in question takes place at temperatures far above *T_g_* = 52° C.

Another point of interest in this and other connections is that *T_g_* is essentially invariant with *x* between *x* ≅ 0.4 and *x* ≅ 0.8 [2], showing that the structure of much of the supercooled liquid state does not undergo any serious change as stage 2 progresses. This indicates that any diffuseness in the degree of crystallinity scale resulting from changes in the supercooled liquid state with increasing crystallization must be rather small if not negligible. The nature of the amorphous material between the crystallites at low *x* values may be slightly different than it is at *x* ≥ 0.4, where some of the molecules are in a strained state. Thus, *T_g_* may be somewhat lower than 52° C in the range 0<*x*<0.4 However, the density difference between amorphous material in the two ranges of *x* mentioned can hardly be very great, as is evidenced by the fact that the specific volume of the pure glass or supercooled liquid obtained by analysis of two “impinged” semicrystalline samples where *x* = 0.39 and *x* = 0.82 [2] was not only reasonable, but also fully in consonance with the specific volumes obtained on extremely thin films that had been strongly quenched to a practically amorphous condition. This provides strong evidence for the approximate validity of the crystallinity scale over the entire range of use. In any event, most of the essential results quoted in this paper are relatively insensitive to the absolute crystallinity scale, and even those that do depend on it, such as *x_m_*, are almost certainly not substantially in error.

## 5. Theory

### 5.1. Homogeneous Nucleation Rate

Using the Eyring theory of absolute reaction rates [18], Turnbull and Fisher [19] have shown that the steady-state rate of homogeneous or primary nucleation *I*, in a condensed system may be expressed in the form

$$I=\frac{NkT}{h}e^{-\Delta F_{p}^{*}/kT}e^{-\Delta \phi_{p}^{*}/kT},$$ (6)

where $\Delta F_{p}^{*}$ is the free energy of activation for transport of molecules a short distance to the surface of the embryo or nucleus, $\Delta \phi_{p}^{*}$ the free energy of formation of a nucleus of critical size, *k* is Boltzmann’s constant, *N* is Avogadro’s number, and *T* the absolute temperature. The quantity $J=(kT/h)\exp(-\Delta F_{p}^{*}/kT)$ may he regarded as the jump rate of the elementary transport process at the supercooledliquid—nucleus interface. The free energy of activation describing this jump rate may be divided into its entropy and enthalpy of activation parts in the usual way giving

$$I=\frac{NkT}{h}e^{\Delta S_{p}^{*}/k}e^{-\Delta H_{p}^{*}/kT}e^{-\Delta \phi_{p}^{*}/kT}.$$ (7)

The objective of this section is to calculate $\Delta \phi_{p}^{*}$ for a linear homopolymer in an appropriate manner, and to indicate certain restrictions that may apply to this quantity. Two types of nuclei relevant to the problem of homogeneous nucleation in linear homopolymers will be considered in detail. The first of these deals with a cylindrical nucleus of radius *r* and length *l*, where there is no restriction on either *r* or *l*. The theory given for this model will prove to apply between *T_c_*, and *T_m_*, where *T_c_* is not too far below *T_m_.* The temperature interval *T_c_<T<T_m_* is called *region A.* The second model deals with a cylindrical nucleus of fixed length *l*_0_ and variable radius *r.* The theory given for this case is applicable between *T_cc_<T<T_c_*, where *T_cc_* is a temperature estimated to be well below *T_m_.* This temperature interval is denoted *region B.* Finally, brief mention is made of the properties of a nucleus of fixed length *l*_0_, and a radius of molecular dimensions, *r*_0_. This model applies below *T_cc_, region C.* The derivations are given in somewhat more detail than is customary in order to clearly bring out the nature of the approximations used. In particular, consideration is given to the possible effect of edges, and to the relative importance of the two surface free energies encountered.

#### Region A: no restriction on length or radius of nucleus

**Figure 7:**
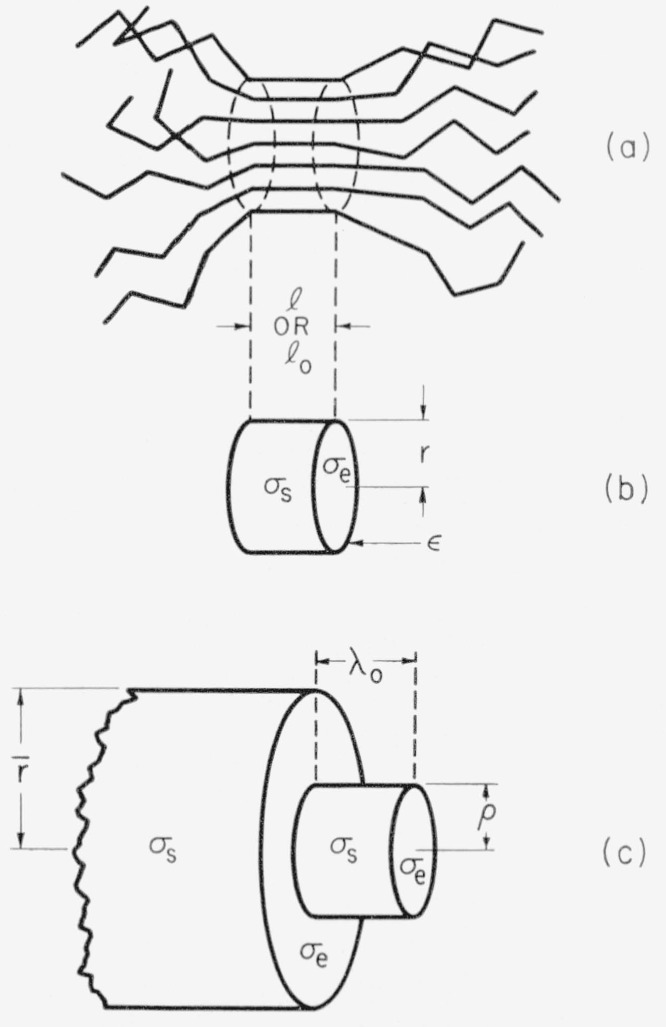
Models of the primary and secondary nuclei.
Schematic diagram showing orientation of polymer chains in primary (homogeneous) nucleus.Model of primary nucleus of radius *r* and length *l* (region A) or *l*_0_ (region B).Model of secondary (growth) nucleus of radius *ρ* and length λ_0_ on end surface of crystallite of radius γ.

We choose as our model for the calculation of $\Delta \phi_{p}^{*}$ a cylindrical nucleus of length *l* and radius *r*, where the polymer molecules are assumed to be normal to the radius (see figure 7a and 7b). The chain molecules are assumed to be normal to the radius because this is the most reasonable manner for a bundle-like nucleus to form spontaneously from an array of linear molecules. In constructing the nucleus in this manner for a bulk phase, we follow the general ideas used by previous workers [5,6]. We have deliberately not proposed a nucleus involving folding of a single chain back on itself forming a loop, since the formation of such a nucleus is in our estimate very likely energetically less favored for a bulk phase than the type proposed; nuclei involving folding of chains would seem more appropriate for the case of extremely dilute solution where only segments of a single polymer chain are apt to be involved in the primary nucleation event. An analysis of the properties of nuclei that start with chain folding will be given in a subsequent publication.

Certain energy quantities are needed to describe the work required to form a cylindrical nucleus. The surface free energy on the lateral surface of the nucleus is *σ_s_*, and the surface free energy on the end is denoted *σ_e_*.^6^ The quantity Δ*f* is defined as the bulk free energy of fusion of crystal per unit volume. *(σ_s_* and *σ_e_* are in erg cm^−2^, and Δ*f* is in erg cm^−3^). Further, we define a quantity *ϵ* as the circumferential residual edge free energy, expressed in erg cm^−1^. Although *ϵ* will prove not to play a significant role in the particular data to be analysed, circumstances may conceivably arise where the effect of *ϵ* will become noticeable. The consideration of effects arising from the edge phase is theoretically justified, since in a system consisting of an aggregate of linear chains, it is simply not possible to construct a nucleus of the required type that contains no edge, or a contour that acts like an edge. Note especially that *ϵ*, as defined here, is not the work required to build 1 cm of edge phase, ℰ, but is instead a residual quantity that depends on certain differences that would reflect any unusually large values of *ℰ* (or *W*_3_).^6^ The residual edge free energy is thus introduced in a manner that does not complicate the customary definitions of either *σ_s_* or *σ_e_* and which permits it to be ignored under many circumstances.

For the above model, the free energy of formation of the nucleus from the supercooled mother phase is

$$\Delta \phi_{p}=2\pi rl\sigma_{s}+2\pi r^{2}\sigma_{e}+4\pi r\epsilon -\pi r^{2}l\Delta f.$$ (8)

The free energy of activation at the saddle point in the free energy surface described by eq (8) is obtained by setting the partial derivatives (∂Δ*ϕ_p_/*∂*r)_l_* and (∂Δ*ϕ_p_/*∂*l)_r_* equal to zero. In this manner there is obtained

$$r^{*}=\frac{2\sigma_{s}}{\Delta f}$$ (9)

for the critical radius, and

$$l^{*}=\frac{4\sigma_{e}}{\Delta f}+\frac{2\epsilon }{\sigma_{s}}$$ (10)

for the critical length of the primary nucleus. Inserting eq (9) and (10) into (8), it is found that

$$\Delta \phi_{p}^{*}=\frac{8\pi \sigma_{s}^{2}(\sigma_{e}+\epsilon \Delta f/\sigma_{s})}{\Delta f^{2}}$$ (11)

The value of Δ*f* in a system with a glass transition has been shown [7] to be accurately described by the relation

$$\Delta f=(\Delta h_{f}\Delta T/T_{m})(T/T_{m}),$$ (12)

where *T_m_* is the equilibrium melting temperature, Δ*h_f_* the heat of fusion at *T_m_* in erg cm^−3^, and Δ*T* the number of degrees the material is supercooled, *T_m_–T.* The usual expression employed in this connection lacks that extra factor *T/T_m_*, which arises from a detailed consideration of the fact that the heat of crystallization must drop below the value Δ*h_f_* as the degree of supercooling is increased. Combination of eq (12) with eq (11) yields the result

$$\Delta \phi_{p}^{*}=\frac{8\pi T_{m}^{4}\sigma_{s}^{2}(\sigma_{e}+\epsilon \Delta f/\sigma_{s})}{\Delta h_{f}^{2}T^{2}\Delta T^{2}}.$$ (13)

The question now arises concerning the relative importance of the terms *ϵ*Δ*f/σ_s_* and *σ_e_* in eq (13). This problem can readily be resolved by noting that

$$|\epsilon |\sim d\sigma_{e}$$ (14)

where *d* is the distance between the chains in the crystal (see footnote 6). Thus, eq (13) may be written in the form $\Delta \phi_{p}^{*}=8\pi T_{m}^{4}\sigma_{s}^{2}\sigma_{e}[1+O(d\Delta f/\sigma_{s})]/[\Delta h_{f}^{2}T^{2}\Delta T^{2}]$. Noting that Δ*h_f_* is about 10^9^ erg cm^−3^ for many polymers, and taking *d* = 5×10^−8^ cm and *σ_s_* = 10 erg cm^−2^, both reasonable values, it is readily determined that the term *d*Δ*f/σ_s_* comes to approximately Δ*T*/100, which will be negligible compared to unity if Δ*T* is small. Thus, the term containing *ϵ* in eq (13) will be unimportant near the melting point, but could have an effect at a moderate degree of supercooling. However, as will be demonstrated shortly, it is probable that even for moderate supercooling a restriction on *l* will have already entered, causing $\Delta \phi_{p}^{*}$ to take on an entirely different form. Thus, in the temperature range where *r* and *l* may be regarded as unrestricted, the expression

$$\Delta \phi_{p}^{*}≅\frac{8\pi T_{m}^{4}\sigma_{s}^{2}\sigma_{e}}{\Delta h_{f}^{2}T^{2}\Delta T^{2}}$$ (15)

gives the free energy of the activated state of the primary nucleus to an acceptable approximation. This result differs slightly from that usually given for the same case [6], because we have here used eq (12) for Δ*f*, rather than the less precise expression Δ*f*≅Δ*h_f_*Δ*T/T_m_*.

Equation (15) may be expressed in the form $\Delta \phi_{p}^{*}=v^{*}\Delta f/2$ where v*, the volume of the nucleus in the activated state, is 16 *πσ_s_*^2^*σ_e_*/Δ*f*^3^.

At this point it is convenient to comment on the anticipated magnitude and behavior of *σ_s_* and *σ_e_.* The lateral surface free energy, *σ_s_*, should be distinctly larger than *σ_e_* for a model where the polymer chains are perpendicular to the radius of the nucleus as indicated in figure 7a. The external environment of the chains on the lateral surface will be that of segments of other molecules in a disordered supercooled liquid phase, while the internal environment will be similar to that of a nonpolymeric molecular crystal possessing a relatively high degree of order. Thus, it is to be anticipated that *σ_s_* will have a relatively normal value, corresponding roughly to that of a nonpolymeric molecular crystal of the same chemical type. Reasonable values of *σ_s_* would thus ordinarily lie in the range 5 to 50 erg cm^−2^. On the other hand, as the environment of the segments is traced along the *l* direction from the crystal proper out through the end, only a relatively small decrease in order will be noticed. The result is that we must commonly anticipate the condition *σ_s_>>σ_e_.* However, it is physically impossible for *σ_e_* to be identical to zero, since this would imply that there was no difference in free energy between the end of the nucleus and the chains adjacent to it. A molecular theory of *σ_e_* would very likely have to take into account chain stiffness. Another point concerning the surface free energies is that they may actually be slightly temperature dependent. If the crystalline state tends to become disordered with rising temperature, a slight concommitant decrease of *σ* is to be expected, but an analysis carried out in the customary way assuming that *σ* is constant should still be quite accurate. The intervention of a firstorder crystal-crystal phase transition below *T_m_*, would, of course, demand special treatment.

The rate of homogeneous nucleation, that applies in region A, where there is no restriction on *r* or *l*, may be obtained by substituting eq (15) into eq (7). Thus,

$$\begin{array}{l} I_{A}=\frac{NkT}{hM{\bar{V}}_{l}}e^{\Delta S_{p}^{*}/R}e^{-\Delta H_{p}^{*}/RT}e^{-8\pi T_{m}^{4}\sigma_{s}^{2}\sigma_{e}/\Delta h_{f}^{2}T^{2}\Delta T^{2}kT}\\ \;(T_{c}<T<T_{m}) \end{array}$$ (16)

where *I_A_* is in nuclei sec^−1^ cm^−3^. In this expression ${\bar{V}}_{l}$ is the specific volume of the supercooled liquid, and *M* the molecular weight of the crystallizing segment. $\Delta S_{p}^{*}$ is expressed in cal mole ^−1^ deg ^−1^, and $\Delta H_{p}^{*}$ in cal mole ^−1^. For this model, r* is 2*σ_s_/*Δ*f*, and to a sufficient approximation, l* is 4*σ_e_*/Δ*f*. Observe that the nucleation term is of the form exp (−*α/T*^3^Δ*T*^2^), where *α* is a constant. An estimate of *T_c_* will be given below.

#### Region B: length restricted to l_0_, radius unrestricted^7^

The calculation of $\Delta \phi_{p}^{*}$ for the case where there is a physical restriction on the length of the nucleus, so that it is a constant, will now be considered. Before proceeding, it will be indicated why consideration of such a model is deemed necessary for a linear polymer. The critical length of the nucleus with variable *r* and *l* is, to the approximation indicated above, given by 4*σ_e_*/Δ*f*. As a result, l* will decrease approximately as 1/Δ*T* as the crystallization temperature is lowered. Now the smallest conceivable length that could be incorporated into a nucleus in an elementary process would be ca. 2.5×10^−8^ cm, which corresponds to the 
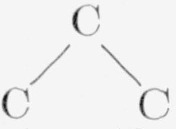
 repeat distance in the zig-zag polymer chain. Alternatively, the unit of crystallization may be larger, and contain a number of such units. Either way, a certain irreducible nucleus length, *l*_0_, is certain to exist. At a temperature *T*_c_, corresponding to a degree of supercooling ΔT_c_, l* will have fallen to a value of *l*_0_. It is found using eq (10) and (12) that

$$\Delta T_{c}≅\frac{4\sigma_{e}T_{m}}{\Delta h_{f}l_{0}}$$ (17)

to a sufficient approximation. At greater degrees of supercooling, eq (15) will no longer be valid, and a revised theory that takes into account the fixed length, *l*_0_, must be used. An example will serve to show that *T_c_* may be close to *T_m_.* Taking *l*_0_ = 10×10^−8^ cm, *T_m_* = 500° K, *σ_e_* = 0.5 erg cm^−2^ and Δ*h_f_* = 10^9^ erg cm^−3^, Δ*T_c_* is found to be about 10° C. The transition between regions A and B will, of course, not be completely abrupt.

Consider now the calculation of the free energy of formation of a nucleus with fixed length and unrestricted radius. Let the length of the nucleus be and let *σ_s_* and *σ_e_* have the same significance as before. (The residual edge free energy *ϵ*, as defined for the model with unrestricted *r* and *l*, may be ignored.) The free energy of formation is

$$\Delta \phi_{p}=2\pi rl_{0}\sigma_{s}+2\pi r^{2}\sigma_{e}-\pi r^{2}l_{0}\Delta f,$$ (18)

from which it is determined by setting *d*Δ*ϕ_p_/dr* = 0 that

$$r^{*}=\frac{l_{0}\sigma_{s}}{l_{0}\Delta f-2\sigma_{e}}.$$ (19)

This leads to

$$\Delta \phi_{p}^{*}=\frac{\pi {\left(l_{0}\sigma_{s}\right)}^{2}}{l_{0}\Delta f-2\sigma_{e}}.$$ (20)

If *σ_e_* is as small as anticipated, *l*_0_Δ*f* will generally considerably exceed 2*σ_e_*, especially at the degree of supercooling that corresponds to crystallization temperatures below *T_c_.* (The minimum value of *l*_0_Δ*f* in region B, which is just at *T_c_*, is 4*σ_e_*). Thus, at temperatures ranging from a little below *T_c_* to considerably lower temperatures, eq (20) may be approximated as $\Delta \phi_{p}^{*}≅\pi l_{0}\sigma_{s}^{2}/\Delta f,$ which, on combination with eq (12), yields

$$\begin{array}{l} \Delta \phi_{p}^{*}≅\frac{\pi T_{m}^{2}l_{0}\sigma_{s}^{2}}{\Delta h_{f}T\Delta T}.\\ \left(T_{cc}<T<T_{c}\right) \end{array}$$ (21)

To the approximation indicated, r* is given by *σ_s_/*Δ*f*. Except for the extra factor *T_m_/T*, eq (21) is the same as that derived by Kahle and Stuart [20], who arbitrarily assumed that *σ_e_* was zero.

It should be remarked that eq (21) in no way implies that *σ_e_* is actually zero, but only that it is small compared to *l*_0_Δ*f*. There is no fundamental objection from either a theoretical or experimental standpoint to the proposition that *σ_e_* may be considerably less than either *σ_s_* or *l*_0_Δ*f*.^8^

The rate of homogeneous nucleation in region B is given by

$$\begin{array}{l} I_{B}=\frac{NkT}{hM{\bar{V}}_{l}}e^{\Delta S_{p}^{*}/R}e^{-\Delta H_{p}^{*}/RT}e^{-\pi T_{m}^{2}l_{0}\sigma_{s}^{2}/\Delta h_{f}T\Delta TkT}\\ \;(T_{cc}<T<T_{c}) \end{array}$$ (22)

where *I* is again expressed in nuclei sec^−1^ cm^−3^. Here *M* is the molecular weight of a segment of length *l*_0_. An estimate of *T_cc_* will be given subsequently.

From an experimental standpoint, the important difference between eqs (16) and (22) is that when the length is unrestricted, the nucleation term is of the form exp[−*α/T*^3^Δ*T*^2^], whereas it is of the form exp[−*β/T*^2^Δ*T*] when the length of the nucleus is *l*_0_. This difference can lead to an experimental decision between eqs (16) and (22). In these expressions the constants are $\alpha =8\pi \sigma_{s}^{2}\sigma_{e}T_{m}^{4}/\Delta h_{f}^{2}k$ and $\beta =\pi l_{0}\sigma_{s}^{2}T_{m}^{2}/\Delta h_{f}k$.

#### Region C: length restricted to l_0_, and radius restricted to molecular dimensions

In region B it will be observed that the radius diminishes with lowering temperature as *σ_s_*/Δ*f*, or approximately as 1/Δ*T.* Eventually, then, r* will shrink to molecular dimensions, just as l* did at *T_c_.* We have (somewhat arbitrarily) chosen a nucleus that contains seven polymer chains, i.e., one with six “surface” segments and one “interior” segment, as the smallest that may reasonably be treated as belonging to region B. If the radius of such a nucleus is denoted *r*_0_, it is readily found that the degree of supercooling corresponding to the lower limit of region B, Δ*T_cc_*, is given to a fair approximation by

$$\Delta T_{cc}≅\frac{\sigma_{s}T_{m}}{\Delta h_{f}r_{0}}.$$ (23)

Thus, the calculations given previously for region B apply between the temperatures *T_c_* and *T*_cc_, corresponding to degrees of supercooling of Δ*T_c_* and Δ*T_cc_*, respectively. At temperatures below *T_cc_*, region C-type nucleation will prevail.

A rough estimate of Δ*T_cc_* may be obtained by setting *T_m_* = 500° K, *σ_s_* = 10 erg cm^−2^, Δ*h_f_* = 10^9^ erg cm^−3^, and *r*_0_ = 7.5×10^−8^ cm, the latter corresponding to a polymer crystal with seven segments, each 5 A in diameter. In this case, Δ*T_cc_*≅67° C. The estimates of *T_c_* and *T_cc_* given imply that type B homogeneous nucleation should frequently appear in the temperature range commonly accessible to crystallization studies in polymers.

We turn now to a qualitative discussion of the nature of homogeneous nucleation in region C. It is necessary to emphasize the fact that unlike *l*_0_, *r*_0_ is not to be considered as an “irreducible” value. The quantity *r*_0_ simply denotes the approximate radius of the nucleus of critical size at the onset of region C. Actually, an important characteristic of region C-type nucleation is that as the temperature is lowered further and further below *T_cc_*, nuclei with radii smaller than *r*_0_ will tend to form. Eventually, critical-sized nuclei that contain five, four, or even three segments of length *l*_0_ must be expected. It will be noticed that the smaller of the region C-type nuclei cannot possibly contain a central molecule, and may therefore be regarded, at least in a certain sense, as partaking mainly of the nature of surface states. The “radius” of such small nuclei is, of course, ill-defined.

An important characteristic of region C is that the rate of nucleation in this region, *I_C_*, will be more rapid than would be expected from an extrapolation from region B. The excess primary nucleation rate in region C is a result of small embryos present in the liquid state that are converted to small nuclei when the liquid is cooled near or below *T_cc_.* This effect will lead to an enhanced crystallization rate in region C, and deserves brief discussion. At any temperature *T*_1_ in the liquid state, the free energy of formation of an embryo always increases as its size increases, in contrast to the case of embryos in a supercooled liquid where the free energy of formation goes through a maximum so that the embryos can become nuclei, and finally stable crystallites. Nevertheless, numerous small embryos (triads, tetrads, etc.) will exist in the superheated liquid above *T_m_*, and the population of such nuclei can be estimated by straightforward methods. (In the expressions for Δ*ϕ*, Δ*T* simply changes sign above *T_m_*.) Now when a specimen is rapidly cooled from a superheating temperature *T*_1_ to a temperature *T*_2_ in the supercooled state, these small embryos will persist. Those that are already the size of nuclei stable at *T*_2_ will in fact represent a source of growth centers at *t* = 0, and those that are subcritical in size will provide a ready source of critical-sized nuclei after some growth. The effect of the presence of such embryos will be negligible in region B since the nuclei necessary here are sensibly larger than the embryos carried down from *T*_1_. Near and below *T_cc_*, however, our calculations indicate that the effect of such embryos will considerably enhance the rate of nucleation. The injection of these embryos will lead to rapid crystallization in the upper part of region C. This phenomenon is aptly described as a “nucleative collapse” of the supercooled liquid state. The authors express the opinion that this phenomenon may sometimes be the underlying cause of the difficulty commonly encountered in preparing amorphous samples of certain crystallizaable polymers by anything but the most rapid quenching. At temperatures sufficiently far below *T_cc_*, the rate of nucleation will have a positive temperature coefficient, since here the principal deterrent to the growth of the small embryos will be the jump rate at the supercooled-liquid—nucleus interface. The transition between region B and C will almost certainly not be completely abrupt.

Finally, it is pointed out that the heat of crystallization, Δ*h*, in region C will be considerably smaller than it is near the melting point as a result of the fact that this quantity must fall below Δ*h_f_* in a glassforming system [7].

**Figure 8:**
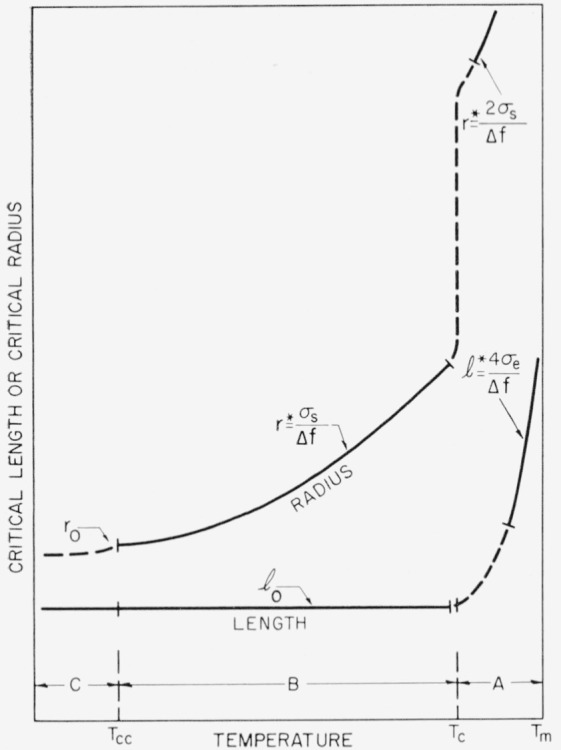
Schematic diagram of radius and length of primary nucleus in activated state as function of temperature. See text for details.

A schematic diagram showing the general type of behavior exhibited by the radius and length of the critical-sized primary nucleus in regions A, B and C is shown in figure 8. The dashed lines on the l* curve in region A indicate the temperature range where effects due to the discrete character of the crystallizing segments of length *l*_0_ may be expected. The dashed line in region A on the r* curve denotes the range where the transition from r* = *σ_s_/*Δ*f* to r* = 2*σ_s_/*Δ*f* is not actually smooth as a result of the “quantized” nature of *l* in the same region. Details of this part of region A have not been given in this paper. The dashed line in the r* curve in region C is intended to denote the “radius” of the various types of nuclei that will form in that region. The fundamental reason *T_cc_* is well below *T_c_* is that *σ_s_* is considerably greater than *σ_e_*, with the result that l* reaches molecular dimensions prior to r*.

In the case where *σ_e_* is larger than we had previously envisioned, and takes on a value where *σ_e_* is close to *l*_0_*σ_s_*/2*r*_0_, region B would be entirely absent. Then the system would exhibit type A primary nucleation down to a transition temperature where the type C initiation would prevail. While such an effect is certainly theoretically possible, it is believed that *σ_e_* will rarely be so large as to completely eliminate region B.

### 5.2. Jump Rate at Supercooled-Liquid—Nucleus Interface

Before proceeding to calculate *G*(*T*) and *Z*_2_(*T*), comment on the validity in the present application of the jump rate at the liquid-nucleus interface, $J=(kT/h)\exp(\Delta S_{p}^{*}/k-\Delta H_{p}^{*}/kT)$, that appears in eq (6) and (7), seems appropriate. In the theoretical development of Turnbull and Fisher, it was explicitly pointed out that this jump rate referred to short- range diffusion of atoms or molecules over a distance on the order of magnitude of a few Angstrom units. It will be noted that the form of the jump rate employed by Turnbull and Fisher is the same as that commonly written for the diffusion of an atom or molecule from one site to another in a crystal. In adopting this form of *J* in the present application, it is thus implicity assumed that the jump rate of polymer segments at the interface is of a crystal-like character. Although little is actually known of the elementary interfacial transport process for either ordinary or polymeric crystals, this appears to be a reasonable approach. If the crystal-like approach is correct for a polymer, it is to be expected, at least over a short temperature range, that $\Delta S_{p}^{*}$ and $\Delta H_{p}^{*}$ will behave as if they are independent of temperature.

It is of interest to indicate in the case of a polymer what the approximate form of the jump rate would be if the viscosity of the supercooled liquid phase controlled the interfacial transport process. According to the simplified but useful approach mentioned by Fox, Gratch, and Loeshak [22], the segmental jump rate in a supercooled linear polymer may be represented as *J_l_* = *J*_0_*P_f_P_E_*, where *J*_0_ is a frequency factor, *P_f_* the probability that a segment has sufficient free volume to jump, and *P_E_* the probability that the segment has sufficient energy to jump. If we use the fractional free volume as defined by Doolittle [23], we may write $P_{f}=\exp[-{\bar{V}}_{0}/({\bar{V}}_{l}-{\bar{V}}_{0})]$, where ${\bar{V}}_{0}$ is the specific volume of the glassy state at 0° K,^9^ and *V_l_* the specific volume of the supercooled liquid or glassy state. *P_E_* may be written in the customary form exp[−E*/RT] where E* is a heat of activation [22]. Thus, *J_l_* may be written as $J_{0}\exp[-{\bar{V}}_{0}/({\bar{V}}_{l}-{\bar{V}}_{0})]\exp[-E*/RT]$. By making use of the remarkable viscosity expression discovered by Williams, Landel, and Ferry [24], which holds from *T_g_* to *T_g_+* 100°, together with the concept that *η* = const./*J_f.E_* [22], where *η* is the macroscopic viscosity, it is easy to show that *P_f_* may be cast in the form exp[−4.12×10^3^/R(51.6+T−*T_g_*)]. *T_g_* is the glass transformation temperature. (The constant 4.12×10^3^ is presumably in calories per mole of kinetic segment.) Thus, near and above *T_g_*, *J_l_∝J*_0_ exp[−4.12×10^3^/R(51.6+ *T*−*T_g_*)] exp[−E*/RT]. E* is a specific property of a given polymer, but the main term, *P_f_*, contains constants that apply generally to glass-forming systems.^10^ The important point here is that the supercooled liquid jump rate is extremely temperature dependent, and becomes very small near and below *T_g_.* This is illustrated by the fact that the overall apparent activation energy of the jump rate calculated as E*_app._ ≡ R∂ln*J_l_/∂*(1*/T*) is 4.12×10^3^*T*^2^/(51.6+*T–T_g_*,)^2^+E*, a quantity that becomes markedly greater as *T* diminishes. This is in sharp contrast to the behavior anticipated for the crystal-like model, which treats $\Delta H_{p}^{*}$as a constant. If data indicated that *J_l_* should be used in eq (6) and (7) instead of *J*, the implication would be that the segmental jump rate in the liquid played an important role in determining the effective jump rate at the interface. It is emphasized that the expression given for *J_l_* is highly approximate.

The particular form of the jump rate used in eq (6) and (7) is actually fairly unimportant in the region where the temperature coefficient of the rate of crystallization is strongly negative, i.e., where the effect of a nucleation term such as exp (−*β/T*^2^Δ*T*) or exp(−*α/T*^3^Δ*T*^2^) is dominant. At some lower temperature, the jump rate term finally overwhelms the nucleation term, and causes the temperature coefficient of the rate of nucleation to swing strongly positive. It is in this low-temperature region that the jump rate essentially controls the rate of crystallization, and where it would be important to know its precise form. For the present, it is assumed that the expression based on the crystal-like model of the interface, $J=(kT/h)\exp(\Delta S_{p}^{*}/k-\Delta H_{p}^{*}/kT)$, is adequate in the temperature interval where the temperature coefficient of the crystallization is negative.

### 5.3. Lineal Growth Rate

The original conception of two-dimensional surface nucleation is due to J. Willard Gibbs. Problems in this category were treated in some detail by Volmer [25], who specifically postulated that crystal growth in low molecular weight materials was a result of surface nucleation. The rate at which the crystallites grow along the *l* direction in the present case will be treated from this standpoint, and ample evidence given subsequently to substantiate this approach. For the sake of clarity, greek symbols are used to denote the radius and length of the growth nucleus.

The critical-sized primary nucleus discussed in previous sections is subject to two types of growth: radial, and that which takes place along the *l* direction. It is clear from the seed crystal experiments with *n* = 1 that the growth process is onedimensional, and must therefore refer to that which takes place in the *l* direction. If the major part of the crystallinity had been introduced into the system by radial growth of disk-like objects, then a “seed crystal” experiment would have yielded *n* = 2 rather than *n* = 1. Two other facts are now introduced. The first is that the free energy surface is such that an increase of *l*, no matter how great, will not lead to an increase of stability of a primary nucleus if it has a radius of r*. Thus we know that the actual growth center formed from the primary nucleus of critical size has a radius that must be larger than r*. A more detailed theoretical analysis indicates that $\bar{r}$ must be at least several times larger than r*. The second fact is that X-ray results, to be introduced later, suggest that $\bar{r}$ is probably within a factor of two of a hundred A. The conclusion that may be drawn from the above is as follows. Once the critical size of the primary nucleus is attained, radial growth up to a mean radius *r*, where *r* is considerably greater than r*, takes place in a time that is extremely short compared to the generation time *τ* of the critical-sized primary nucleus. Thus, the actual homogeneously formed nucleation center that is effective in the system has a radius̄, and a length l* or *l*_0_, the latter depending on whether the primary nucleation takes place in region A or B. The cessation of the rapid radial growth (which is almost certainly nucleation controlled) may be regarded as being a result of edgewise impingements or volume strain. Calculations from impingement theory [16] render it highly likely that edgewise impingements of thin disks will be relatively effective even at a very low degree of transformation; only an insignificant amount of crystallization will result from the edgewise growth of sufficiently thin disks. It is emphasized that the rate-determining step in the formation of the actual nucleation center of radius *r* is the formation of the critical-sized nucleus of radius r*. The *n* = 2 isotherms are a result of the formation of growth centers of radius $\bar{r}$ and length l* or *l*_0_ at uniformly later and later dates, and the subsequent one-dimensional growth at a rate *G* in the *l* direction.

As indicated above, the rapid growth of the primary nucleus in the radial direction, *G_r_* = *dr/dt*, is almost certainly nucleation controlled. Its rapidity suggests that the secondary radial growth nucleus is comparatively easy to form in the temperature range of interest here. At sufficiently high temperatures, *G_r_* could become slow enough to fall in the measurable range. No more need be said of *G_r_* for the present, since it does not directly lead to detectable amounts of crystallization in the particular case that we will consider.

Consideration will now be given to a simple model that describes *G* = *dl/dt*, the rate of growth in the *l* direction. It is assumed that a secondary or growth nucleus of radius *ρ* and a fixed length λ_0_ forms on the end of the crystallite as shown in figure 7(c), and that this is the rate-determining step in the lineal growth rate. As soon as the growth nucleus is formed, the layer of length λ_0_ is quickly completed by radial growth on the face of the crystallite. The lineal growth process then continues through the formation of a new growth nucleus. The fixed length λ_0_ is, of course, numerically identical to the *l*_0_ used in the discussion of homogeneous nucleation, but this identity will not be employed for the time being.

The free energy of formation of the growth nucleus is

$$\Delta \phi_{g}=2\pi \rho \lambda_{0}\sigma_{s}-\pi \rho^{2}\lambda_{0}\Delta f.$$ (24)

Observe that no term involving the free energy of formation of the end of the growth nucleus is included in eq (24). This results from the fact that the total area of end for the entire crystallite is the same before as after the formation of the growth nucleus. Thus, eq (24) in no way implies that *σ_e_* is zero. Note further that no term in *ϵ* is included. This results from the fact that the residual work involved in building the outermost convex edge of the secondary nucleus, 2*πρϵ*, will almost exactly compensate the residual −2*πρϵ* involved in forming the concave “edge” at the nucleus-crystallite interface. Even without such compensation, the effect of *ϵ* on the result would be completely negligible. By taking the derivative of eq (24) with respect to *ρ* and setting the result equal to zero, it is found that critical radius of the growth nucleus is

$$\rho^{*}=\sigma_{s}/\Delta f,$$ (25)

and on inserting this into eq (24), it is found that the free energy of formation of a growth nucleus of critical size comes to

$$\Delta \phi_{g}^{*}=\frac{\pi \lambda_{0}\sigma_{s}^{2}}{\Delta f}.$$ (26)

As before, Δ*f* is given by eq (12). The rate of formation of the growth nuclei will depend on an equation of the form of eq (7), except that the pre-exponential factor is treated in a different way. Turnbull [26] has shown that the pre-exponential term is in this case *λ*_0_*kT/h.* Hence, the lineal growth rate in cm sec^−1^ is given by

$$G=2\frac{\lambda_{0}kT}{h}e^{\Delta S_{g}^{*}/R}e^{-\Delta H_{g}^{*}/RT}e^{-\pi T_{m}^{2}\lambda_{0}\sigma_{s}^{2}/\Delta h_{f}T\Delta TkT}$$ (27)

where $\Delta S_{g}^{*}$ and $\Delta H_{g}^{*}$ are the entropy and enthalpy of activation, respectively, of the elementary short- range transport process at the growth-nucleus—crystal interface. The factor of 2 in the preexponential was inserted on the assumption that each crystallite can grow along both the +*l* and −*l* directions.

The growth nuclei will not show effects in the vicinity of the A→B transition such as occurred for the primary nuclei at *T_c_*, since λ_0_ is fixed in both regions A and B. Hence eq (27) is valid in regions A and B for a polymer that has an *n* = 2 isotherm resulting from sporadic initiation of centers that grow in the *l* direction. At T_cc_, ρ* will have fallen to a value *ρ*_0_, which is numerically equal to *r*_0_. Thus, in region C, the growth nuclei will tend to resemble surface states, but no growth nuclei will be carried down from above *T_m_.* Thus, the rapid crystallization rate postulated in region C is a result of unusually rapid primary nucleation rather than unusually rapid growth.

On account of the fact that the growth centers in region A will be generated at a greater average distance from each other than in region B or C, threedimensional growth of the sporadically born centers may develop somewhere in region A. (In some cases, three-dimensional growth might begin in the upper part of region B.) This will be discussed in more detail in section 7. If this occurred, *n* would tend toward a value of 4, and the expressions for *G* and *Z* would have to be modified. However, up to the highest temperature studied, poly(chlorotrifluoroethylene) exhibits an *n* value of 2.0, with the result that we need consider only one-dimensional growth in the analysis of the present data. Detailed evidence showing that the indigenous mode of growth at temperatures at and below ~205° C cannot be spherulitic or three-dimensional will be given in section 7.

It should be recognized that $\Delta H_{g}^{*}$ is not necessarily the same as $\Delta H_{p}^{*}$, and a similar observation holds for $\Delta S_{g}^{*}$ and $\Delta S_{p}^{*}$. Also, it is necessary to admit of the following possibilities: (a) the crystallike jump rate may apply to *G*, while that calculated from the free volume theory may apply to *I*, or vice versa; (b) the free-volume jump rate may apply to both *I* and *G.* Nevertheless, the crystal-like approximation, with suitable values of ΔH* and ΔS*, should suffice at high growth temperatures. At low growth temperatures where the nucleation term is no longer dominant, a decision between the various alternatives should be possible, provided that precise rate measurements are obtained over a sufficient range of temperature.

### 5.4. Bulk Crystallization Rate

The rate constant determining the bulk free growth rate, *Z*_2_, is shown in appendix 9.4 for the case of nuclei born at later and later dates that grow in a one-dimensional manner to be

$$Z_{2}=(\pi {\bar{r}}^{2}/2)(V_{l}/V_{c})IG.$$ (28)

Here $\pi {\bar{r}}^{2}$ is the area of the growing face of the crystallite, ${\bar{V}}_{l}$the specific volume of the supercooled liquid, and ${\bar{V}}_{c}$ the specific volume of the crystal. The quantity *Z*_2_ has the dimensions sec^−2^, since *I* is in cm^−3^ sec^−1^, *G* in cm sec^−1^, and $\bar{r}$ in cm.

The bulk crystallization rate constant takes on two distinct forms depending on the type of homogeneous nucleation. In region A, where *r* and *l* are unrestricted, combination of eqs (16), (27), and (28) yields the result

$$\begin{array}{l} Z_{2(A)}=\\ Z_{0}e^{-(\Delta H_{p}^{*}+\Delta H_{g}^{*})/RT}e^{-8\pi T_{m}^{4}\sigma_{s}^{2}\sigma_{e}/\Delta h_{f}^{2}T^{2}\Delta T^{2}kT}e^{-\pi T_{m}^{2}\lambda_{0}\sigma_{s}^{2}/\Delta h_{f}T\Delta TkT} \end{array}$$ (29)

#### (Region A)

where *Z*_0_ is $\pi {\bar{r}}^{2}N\lambda_{0}k^{2}T^{2}/h^{2}M{\bar{V}}_{c}\exp(\Delta S_{p}^{*}/R+\Delta S_{g}^{*}/R)$. On the other hand, in region B, where *l* is restricted to the value *l*_0_ for the homogeneously formed nucleus,

$$Z_{2(B)}=Z_{0}e^{-(\Delta H_{p}^{*}+\Delta H_{g}^{*})/RT}e^{-\pi T_{m}^{2}(l_{0}+\lambda_{0})\sigma_{s}^{2}/\Delta h_{f}T\Delta TkT}$$ (30)

#### (Region B)

where *Z*_0_ is the same as quoted for eq (27). If it is remembered that 4 is numerically equal to λ_0_, the last term in eq (30) may be written in the form $\left(-2\pi T_{m}^{2}\lambda_{0}\sigma_{s}^{2}/\Delta h_{f}T\Delta TkT\right)$.

## 6. Application to poly(chlorotrifluoroethylene)

### 6.1. Preliminary Analysis of Temperature Dependence of Z_2_

It will be observed from eqs (29) and (30) that *Z*_2_ will exhibit a different variation with temperature depending on whether the measurements were taken in region A or region B. If the homogeneous nucleation is of the type postulated for region B, then a plot of log *Z*_2_ against 1*/T*^2^Δ*T* should yield an essentially straight line. On the other hand, if the homogeneous nucleation corresponds to the type postulated for region A, a plot of log *Z*_2_ against 1*/T*^3^Δ*T*^2^ should yield a more nearly straight line than a plot of log *Z*_2_ against 1/*T*^2^Δ*T.* In all cases, the exponential term $\exp[-(\Delta H_{p}^{*}+\Delta H_{g}^{*})/RT]$ will have but little effect in the temperature range where *Z*_2_ has a strongly negative temperature coefficient.

Experimental values of *Z*_2_ and log *Z*_2_ are given in table 3. The values were obtained by analysis of the isotherms shown in figure 2 using eq (4). The time *t* required for the sample to reach a certain degree of crystallinity *x* was measured, and *Z*_2_ calculated from the relation *Z*_2_ = −*t*^−2^ln (1–*x*). This expression was derived using *x_w_* = l. In most cases *x* = 0.15 was used, but in others the value *x* = 0.10 was employed. At such low *x* values, *Z*_2_ proved to be independent of the choice of *x*. The precision of the *Z*_2_ values at *T*_2_ > 180° C is estimated to be about 5 percent, and that at *T*_2_ = 170° C is believed to be better than about 30 percent.

**Figure 9:**
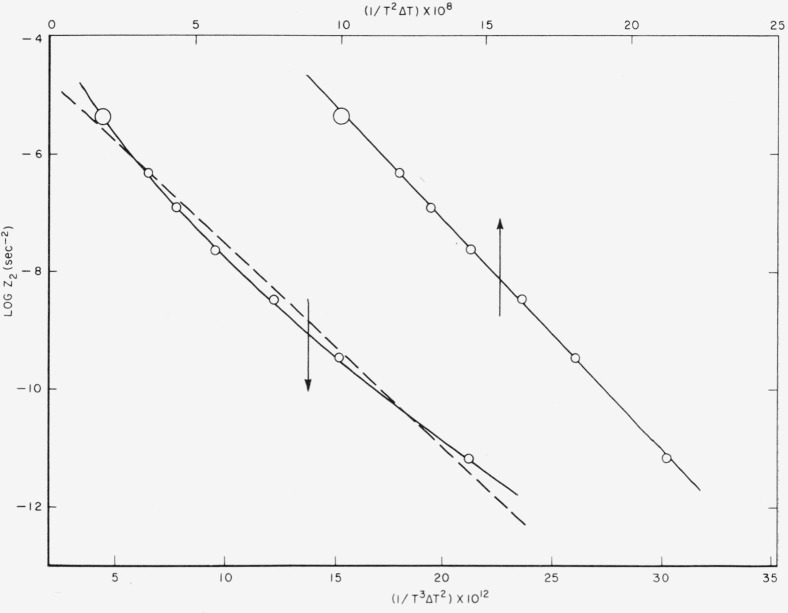
Plot of log Z_2_ versus 1/T^2^ΔT and 1/T^3^ΔT^2^ used in preliminary analysis of Z_2_. The Z_2_ data shown were obtained directly from the *n* = 2 isotherms (see table 3). ΔT was calculated using *T_m_* = 221° C. The dashed line for 1/*T*^3^Δ*T*^2^ shows the best straight line that can be fitted for this type of plot. The solid line for the 1/*T*^2^Δ *T* plot is a least-squares straight line. The best fit is obtained with 1/*T*^2^Δ*T*, showing that the *Z*_2_ data refer to region B.

A plot of log *Z*_2_ against 1*/T*^2^Δ*T* and 1/*T*^3^Δ*T*^2^ is shown in figure 9. The linearity of the 1*/T*^2^Δ*T* plot leaves no doubt that by far the best representation of *Z*_2_ is that given by eq (30). This indicates that *I* is given by eq (22). *Thus, the experimental results pertain to region B homogeneous nucleation, where r is unrestricted and the length of the primary nucleus is l*_0_. This is just what was anticipated from the rough estimates of *T_c_* and *T_cc_.* This result, when considered together with the fact that the growth rate is a nucleation rather than a jump rate controlled process (see below), also clearly indicates that the form of the nucleation term in the expression for *G*, eq (27), is correct with respect to the exponent of Δ*T*.

Before proceeding, it is necessary to dispose of one possibility that presents itself. It will be noticed that if *σ_e_* were extremely small, eq (29) would take on the same form as eq (30), and then lead to the 1/*T*^2^Δ*T*-type dependence found experimentally. This argument for the correctness of eq (29) in the present application is invalid, because such a small *σ_e_* value would be associated with a very small Δ*T_c_* value, and would in any case clearly put the experimental data in region B, i.e., in the range where eq (30) is appropriate.

The finding that eq (30) provides the best description of *Z*_2_ as a function of temperature is not altered by any anticipated uncertainty in *T_m_.* The value of *T_m_* used in calculating Δ*T* was 221° C = 494.2° K. The highest melting point actually observed for a sample of this polymer was *T'_m_* = 218.0° C. This result was obtained on a sample that had been crystallized a long time and to a high degree of crystallinity at a temperature near the melting point, a situation which is conducive to formation of large unstrained crystallites. The value *T'_m_* = 218.0° C is, of course, a lower limit for *T_m_.* The value *T_m_* = 221° C was obtained by a simple extrapolation procedure [12], and is almost certainly correct to within ±3° C.^11^ Even doubling this error does not alter the conclusion that eq (30) rather than eq (29) provides by far the best representation of *Z*_2_(*T*). The basic reasons that a positive decision can be reached between eqs (29) and (30) in this case are that the *Z*_2_ data are reasonably precise, and that the relative error in Δ*T* is small owing to the high degree of supercooling used. Even if *T_m_* is accurately known, it would be difficult to differentiate between the Δ*T*^−1^ and Δ*T*^−2^ type bulk crystallization laws in the A→B transition region.

### 6.2. Detailed Analysis of I, G, and Z_2_

Equation (30) will be used for the detailed analysis of *Z*_2_. In order to obtain the best value of (*l*_0_+λ_0_)*σ_s_*^2^, an estimate of $\Delta H_{p}^{*}+\Delta H_{g}^{*}$ must be made. It has been observed in careful dielectric studies that an activation energy of 16,000 calories mole^−1^ holds for the principal relaxation time in the crystalline phase of poly(chlorotrifluoroethylene) [27]. Nearly the same activation energy also appears to apply to one of the important relaxation times present in the supercooled liquid, suggesting a similar segmental motion in the two phases. For the purposes of calculation it will therefore be assumed that $\Delta H_{g}^{*}=\Delta H_{p}^{*}=16,000\;{\text{cal}\;\text{mole}}^{-1}$, so that $\Delta H_{g}^{*}+\Delta H_{p}^{*}=32,000\;{\text{cal}\;\text{mole}}^{-1}$. With this value, and the value of Δ*h_f_* = 9.10×10^8^ erg cm^−3^ derived from Bueche’s measurement [28] of the heat of fusion of the pure crystalline phase, which was quoted as 10.3 cal g^−1^, it is calculated from eq (30) that

$$\log\;Z_{2}=\log\;Z_{0}-\frac{6996}{T}-\frac{2.651\times 10^{12}(l_{0}+\lambda_{0})\sigma_{s}^{2}}{T^{2}\Delta T}.$$ (31)

Rearranging, it is found that

$$(l_{0}+\lambda_{0})\sigma_{s}^{2}=\frac{T^{2}\Delta T\log Z_{0}}{2.651\times 10^{12}}-\frac{T^{2}\Delta T}{2.651\times 10^{12}}\left[\frac{6996}{T}+\log Z_{2}\right].$$ (32)

**Figure 10:**
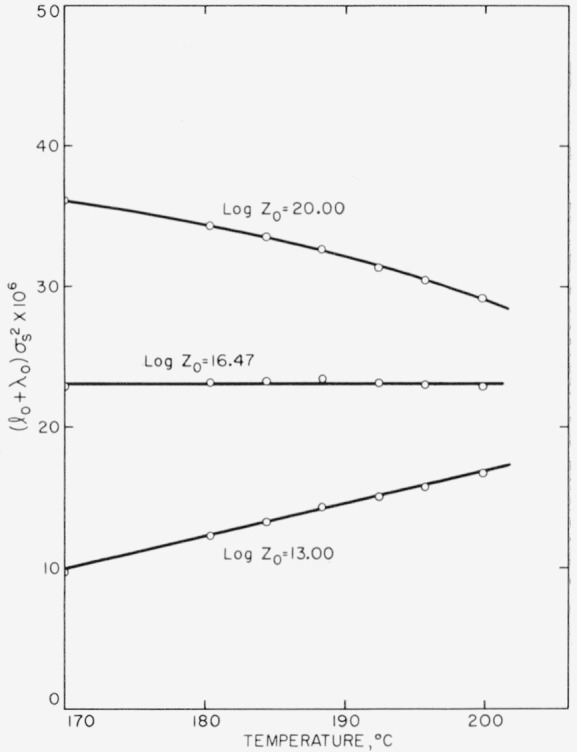
Plots of (l_0_ + λ_0_)σ_s_^2^ as a function of temperature for various assumed values of Z_0_. This plot is used in the detailed analysis of *Z*_2(_*_B_*) to obtain numerical values of log *Z*_0_ and (*l*_0_+λ_0_)*σ_s_*^2^. The value log *Z*_0_=16.47 gives the (*l*_0_+λ_0_)*σ_s_*^2^ value that is most nearly constant with temperature.

According to the arguments given earlier, *σ_s_* should be nearly constant with temperature, so that (*l*_0_ + λ_0_)*σ_s_*^2^ will likewise be constant. The analysis is then carried out by inserting experimental values of log *Z*_2_ into eq (32), and determining the value of log *Z*_0_ that leads to a constant value of (*l*_0_+λ_0_)*σ_s_*^2^ In this manner, it is determined that log *Z*_0_= 16.47 and that (*l*_0_+λ_0_)*σ_s_*^2^=2.31×10^−6^ erg^2^ cm^−3^ (best values obtained by least-squares analysis). Remembering that *l*_0_=λ_0_ this gives *l*_0_*σ_s_*^2^=λ_0_*σ_s_*^2^=1.15×10^−6^ erg^2^ cm^−3^. In order to illustrate clearly how the results depend on *Z*_0_, plots of (*l*_0_+λ_0_)*σ*_s_^2^ versus temperature are shown in figure 10 for various values of *Z*_0_.

Except for $\Delta H_{p}^{*}+\Delta H_{g}^{*}$, which had to be estimated, (*l*_0_+λ_0_)*σ_s_*^2^ obtained in this manner depends only on known constants and the experimental data. The value of (*l*_0_+λ_0_)*σ_s_*^2^ is quite insensitive to the choice of $\Delta H_{p}^{*}+\Delta H_{g}^{*}$, and the value quoted above may be considered a “best” value. That $\left(l_{0}+\lambda_{0}\right)\sigma_{s}^{2}$ is insensitive to $\Delta H_{p}^{*}+\Delta H_{g}^{*}$ is illustrated by the fact that an analysis of the slope of the log *Z*_2_ versus 1*/T*^2^Δ*T* plot in figure 9 yields (*l*_0_+λ_0_)*σ_s_*^2^= 1.96× 10^−5^ erg^2^ cm^−3^, corresponding to the assumption $\Delta H_{p}^{*}+\Delta H_{g}^{*}=0$. Use of the Williams-Landel-Ferry (free volume) jump rate would lead to a somewhat higher value of (*l*_0_+λ_0_)*σ_s_*^2^ than that given, but the difference is hardly significant from the standpoint of nucleation theory.

**Figure 11:**
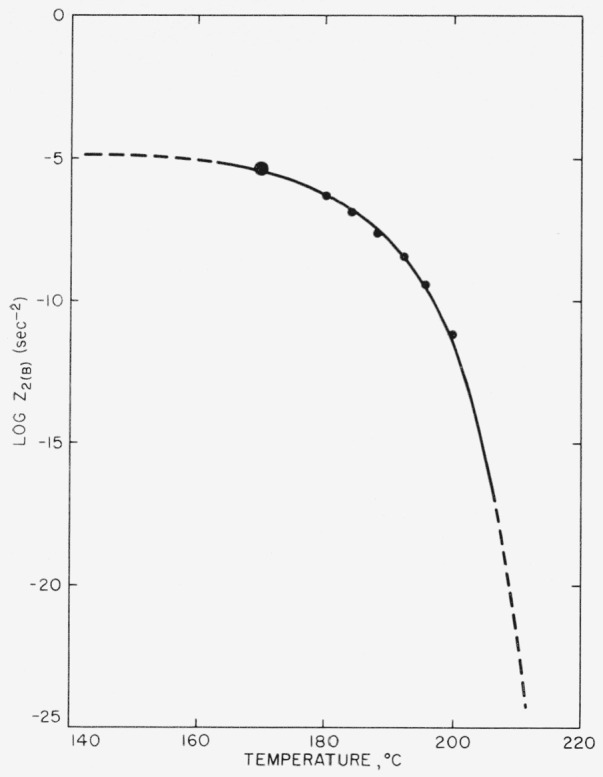
Log Z_2_(_B_) as a function of temperature. Experimental points obtained from *n*=2 isotherms ●, theoretical curve ———. The dashed lines indicate where region A and C type homogenous nucleation may enter.

The fit to the data of the theoretical curve calculated using log *Z*_0_= 16.47, (*l*_0_ + λ_0_)*σ_s_*^2^=2.31 × 10^−5^ erg^2^ cm^−3^, and $\Delta H_{p}^{*}+\Delta H_{g}^{*}=32,000\;{\text{cal}\;\text{mole}}^{-1}$ in eq (30) is shown in figure 11. The agreement is highly satisfactory. The predicted bend off in *Z*_2_ at low temperatures is due partly to the lowering of the jump rate, and partly to the diminution of Δ*f*.

The overall entropy of activation associated with the bulk crystallization mechanism, $\Delta S_{p}^{*}+\Delta S_{g}^{*}$, may be estimated by inserting the numerical value of *Z*_0_ into the theoretical expression for *Z*_0_. If we assume that the crystallizing segment consists of four monomer units, so that *l*_0_=λ_0_=10^−7^ cm and *M* =4×116.5=466 g mole^−1^, and let $\bar{r}=1\times 10^{-6}$ cm, and ${\bar{V}}_{c}=0.473\;{\text{cm}}^{3}g^{-1}$, it is determined that $\Delta S_{p}^{*}+\Delta S_{g}^{*}=-56.8{\;\text{cal}\;\text{mole}}^{-1}\deg^{-1}$. The value of $\bar{r}$ is obtained from the work of Franklin, who has estimated the size of the crystallites using X-ray line- width measurements [29]. In carrying out the analysis, Franklin assumed that the line-broadening was entirely due to crystallite-size effects. This assumption means that the figures quoted are minimum values. A value of $\bar{r}≅200A$ was obtained for a specimen crystallized well into stage 2; the value relevant to stage 1 is undoubtedly smaller (roughly 50A) as Franklin’s work on quenched samples showed. The nominal value $\bar{r}=100A$ was arbitrarily chosen for purposes of calculation. On account of the uncertainty in λ_0_ and $\bar{r}$, as well as the fact that log *Z*_0_ depends on the choice of $\Delta H_{p}^{*}+\Delta H_{g}^{*}$, the calculated value of $\Delta S_{p}^{*}+\Delta S_{g}^{*}$ is certainly not very accurate. Nevertheless, it is considered certain that $\Delta S_{p}^{*}+\Delta S_{g}^{*}$ is strongly negative. This result clearly suggests that for a polymer the activated state in the elementary short-range diffusion mechanism at the interface is less random than the initial state, a not unlikely state of affairs.

It is of interest to estimate the value of *σ_s_.* This requires that *l*_0_=λ_0_ be estimated. If it is assumed that this quantity is 10^−7^ cm or 10A, which corresponds to four monomer units, *σ_s_* proves to be 10.8 erg cm^−2^. This choice of *l*_0_ = λ_0_ is almost certainly within a factor of four of the correct one: the smallest conceivable value would be 2.5A, corresponding to the 
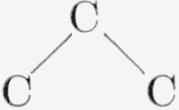
 repeat distance in the chain, while the largest elementary crystallizing unit that need be seriously considered would correspond to the spiral repeat length of 35 to 43A reported for this polymer [30, 31, 32]. Thus, the above estimate of *σ_s_* should be within a factor of two of the true value. Further, the value *σ_s_* =10.8 erg cm^−2^ is quite close to what one would expect for a somewhat disordered halocarbon crystal. Thus, Thomas and Stavely [33] quote *σ* = 13.9 erg cm^−2^ for rotationally ordered carbon tetrachloride, and *σ* = 6.67 erg cm^−2^ for rotationally disordered carbon tetrachloride.

Another point concerning *σ_s_* is that it leads to a reasonable value of ***σ***/Δ*H_f_*, which is the ratio of the free energy of formation of a certain amount of surface phase to the heat of fusion of the same amount of bulk phase [26, 33]. Taking *σ_s_* = 10.8 erg cm^−2^, and assuming that the thickness of the surface phase, *d_s_*, is one molecule or 5.6A [30, 31] thick, ***σ****/*Δ*H_f_* comes to 0.21. The usual value of this quantity for ordinary molecular crystals is about 0.3 [33]. The value of ***σ****/*Δ*H_f_* obtained for poly(chlorotrifluoroethylene) is somewhat higher than that calculated for certain other polymers [6].

A prominent feature of the present data and analysis is that λ_0_*σ_s_*^2^ can be estimated directly from the *n* = 1 seed crystal runs, which are a measure of *G.* This will provide important evidence for the validity of the proposed theory of primary and secondary nucleation.

Some of the *n* = 1 seed crystal experiments were carried out on identically preconditioned samples of polymer that were heated to precisely the same *T*_1_ value, and then crystallized at different growth temperatures. The above mentioned pretreatment introduced the same number and size distribution of seed crystals into each sample. In the case of two specimens where *n*_0_, the number of seed crystals per unit volume, is the same, and where $\bar{r}$ is the same,

$$\frac{Z_{1(i)}}{Z_{1(j)}}=\frac{G_{i}}{G_{j}}=\frac{t_{j}}{t_{i}}.$$ (33)

The subscripts *i* and *j* represent two growth temperatures, *t_i_* and *t_j_* the times required to reach a specified degree of crystallinity at *i* and *j*, and *G_i_/G_j_* the ratio of the lineal growth rate at the two temperatures. Equation (33) is readily derived from eq (A–10) of appendix 9.3, and the properties of eq (4) in the region of superposition.

The method of analysis is illustrated below. There is no way to obtain the absolute value of *G* from specific volume measurements alone, and the treatment used therefore deals with the ratio of *G* values. For purposes of illustration we will deal with the value of *G_i_/G_j_* where *i* = 196.2° and *j* = 203.9° C listed in table 4. The previous history of the samples in pair I was identical, as indicated in the footnote of the table. Equation (27) is used for the analysis of *G*. Denoting the pre-exponential in eq (27) as *G*_0_, and assuming it to be constant, and taking $\Delta H_{g}^{*}=16,000\;{\text{cal}\;\text{mole}}^{-1}$, there is obtained

$$\begin{array}{l} \log\;(G_{i}/G_{j})=3498\left[\frac{1}{j}-\frac{1}{i}\right]\\ +2.651\times 10^{12}\lambda_{0}\sigma_{s}^{2}\left[\frac{1}{j^{2}(T_{m}-j)}-\frac{1}{i^{2}(T_{m}-i)}\right] \end{array}$$ (34)

where *i* = 469.4° K and *j* = 477.1° K. Taking the experimental value of *G_i_/G_j_* from table 4, it is determined from eq (34) that λ_0_*σ_s_*^2^ = 1.02×10^−6^ erg^2^ cm^−3^.

A value of *λ*_0_*σ_s_*^2^ of 1.08 × 10^−5^ erg^2^ cm^−3^ is obtained from the other run listed in table 4, where *i* = 191.5° C and *j* = 205.2° C. The previous history of the two samples was identical, and is described in the footnote of the table. The mean value for the two determinations is *λ*_0_*σ_s_*^2^ = 1.05 × 10^−5^ erg^2^ cm^−3^ which is in good agreement with the value 1.15 × 10^−5^ erg^2^ cm^−3^ obtained from the analysis of *Z*_2_, i.e., from the data on the *n* = 2 bulk crystallization isotherms. The result leaves little doubt concerning the fact that the primary and secondary nuclei in region 13 are energetically nearly equivalent, in accord with the theoretical deductions given in section 5. The value of *λ*_0_*σ_s_*^2^ obtained is very insensitive to the choice of $\Delta H_{g}^{*}$.

**Figure 12:**
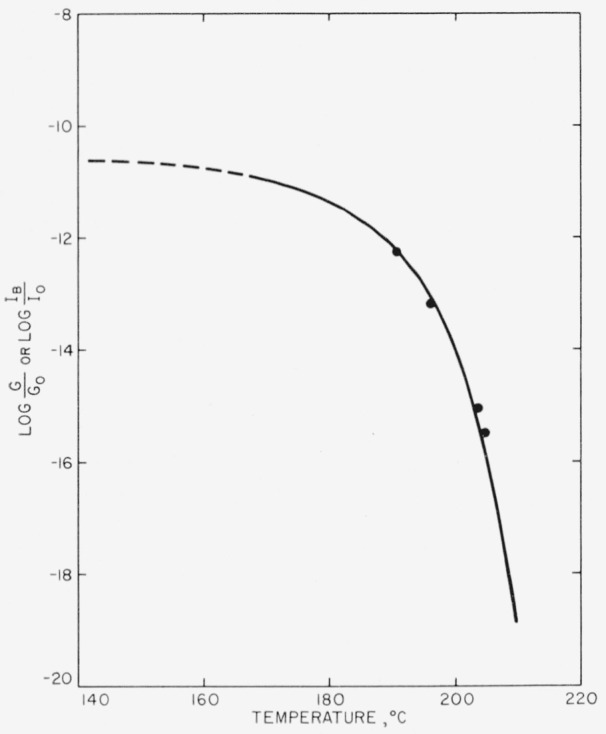
Log I_B_/I_0_ and log G/G_0_ as a function of temperature. The experimental points • refer to *G/G*_0_ values obtained from the *n* = 1 isotherms. The dashed portion of the curve shows where the theoretical curve for *I_B_/I*_0_ may become invalid owing to the onset of region C type primary nucleation.

The ratio *G/G*_0_ can readily be calculated as a function of temperature by inserting the values $\Delta H_{g}^{*}=16,000\;{\text{cal}\;\text{mole}}^{-1}$ and λ_0_*σ_s_*^2^ = 1.15× 10^−5^ erg^2^ cm^−3^ into eq (27). This particular value of λ_0_*σ_s_*^2^ is used, even though obtained from the *Z*_2_ data, since it is based on a larger number of experimental points. The resulting plot of log (*G/G*_0_) versus *T* is plotted in figure 12. The four points shown are those obtained from table 4.

The experimental results obtained with the seeded specimens leave no doubt concerning the fact that *G* has a negative temperature coefficient between 191.5° and 205.2° C, and is therefore nucleation controlled in this region as postulated. This result also certainly holds for growth temperatures somewhat above and well below this range. At least in this case, then, it is well established that it would have been in error to assume that *G* was everywhere diffusion controlled, i.e., possessed a positive temperature coefficient as for $G≅G_{0}\exp(-E_{D}^{*}/RT)$.

The ratio *I/I*_0_ is now determined. It will be observed from eqs (22) and (27) that *I/I*_0_, where $I_{0}=(NkT/hM{\bar{V}}_{l})$, has precisely the same form as *G/G*_0_. If $\Delta H_{p}^{*}$ is set equal to $\Delta H_{g}^{*}$, then the plot shown in figure 12 for log *G/G*_0_ is valid for log *I/I*_0_ as well, and has been so labelled. The plot of log *I/I*_0_ applies in region B only, so *I* = *I_B_.*

It should be realized that the determination of the actual values of *Z*_2_ as a function of temperature, together with our direct knowledge of the relative values of *G* as a similar function, is tantamount to an experimental determination of the temperature dependence of *I.* This is a result of the fact that *Z*_2_ is proportional to *IG.* Thus, the values of log *I/I*_0_ in the interval 191 to 200° C shown in figure 12 may for all practical purposes be regarded as experimentally determined. The error at somewhat higher temperatures, as well as that down to 170° C is probably small. The log *I/I*_0_ versus *T* curve plotted in figure 12 cannot be reproduced even for a restricted temperature interval using eq (16) with *any* choice of *σ_s_*^2^*σ_e_.* Only eq (22), which has a l/*T*^2^Δ*T*-type dependence on temperature, can reproduce the data.

### 6.3. Dimensions of the Nuclei and Crystallites

In region B, the radius of the primary nucleus of critical size is given by r* = *σ_s_/*Δ*f.* Calculations using the previously mentioned value of *σ_s_* give r* = 12.8A at 170° C, and r* = 29A at 200° C. Thus, the activated state contains segments from about 20 polymer chains at 170° C and about 100 chains at 200° C. The critical radius of the secondary nucleus, ρ*, is the same as r*. Remembering that *l*_0_ = λ_0_ ≅10A, both the primary and secondary nuclei of critical size will always have the physical appearance of a flat cylinder, or platelet, since r*>*l*_0_ and ρ*>λ_0_.

The crystallites in poly(chlorotrifluoroethylene) have a “grown” radius in stage 2 of about 200A, as determined by X-ray studies [29]. The mean radius to which the primary nuclei rapidly expand in stage 1 before they begin to grow slowly at a rate *G* along the *l* direction is less than in stage 2. As indicated earlier, $\bar{r}$ is roughly 50A in the early stages of the crystallization, though it must again be pointed out that the estimates of $\bar{r}$ depend on the assumption that the X-ray line-broadening was solely due to crystallite size effects; the existence of disorder in the crystal could cause the estimates of $\bar{r}$ to be too low. In any case, the primary nucleation event- leads to growth centers that are platelets with radii which are nominally 50 to 100A, and with a thickness (called *l*_0_ in our notation) of about 10A. The mean length to which these grow, $\bar{l}$, cannot be accurately determined from the present data, since in the region of superposition,

$$\bar{l}≅\frac{Gt}{2}≅\frac{Gx^{\prime 1/2}}{2Z_{2}^{1/2}},$$ (35)

and absolute values of *G* are not available. It would probably be reasonable to assume that $\bar{l}$ was of the same order of magnitude as $\bar{r}$, but nothing in the present treatment renders this certain. It would appear to be reasonably certain, however, that the length of most of the crystallites does not exceed 500 to 1000A, since otherwise the *n* = 2 samples would scatter more visible light than they do. It is of some interest to note that the predicted shape of the “grown” crystallites in the bulk polymer is in a general sense roughly cylindrical (plate-, drum-, or fiberlike) with the long axes of the polymer molecules normal to the radius, and that this does not depend on special mechanisms such as chain folding. Impingement theory requires a distribution of lengths and radii for the grown crystallites.

When the rate of primary nucleation in regions A and B is considered in relation to the rate of growth, together with the nature of impingements, it is readily seen that the mean size of the “grown” crystallites will be smaller in region B than in region A. Thus, the A→B transition should be accompanied by a decrease in crystallite size that is detectable by X-ray methods. The temperature dependence of the “grown” radius should bear a resemblance to the r* curve in regions A and B shown in figure 8, except of course, that the scale of the abscissa will differ in the two cases. A similar effect would be noted at a B→C transition. If region B is absent, as for *σ_e_* = *l*_0_*σ_s_/*2*r*_0_, a noticeable drop in mean crystallite size would appear at the A*→*C transition. The crystallites formed in region C will be extremely small.

### 6.4. Regions A and C

The present theory of homogeneous nucleation and growth would be strengthened further if it could be shown that nucleation typical of regions A and C existed in poly(chlorotrifluoroethylene).

Definite evidence of an anomaly in the bulk crystallization rate below 170° C ascribable to region C-type nucleation has been obtained. Cooling rate studies carried out with thermocouples embedded in sheets of poly(chlorotrifluoroethylene) indicated that if they are first heated to 305° C and then plunged in ice water, specimens ~2mm thick can be cooled through the temperature interval 170° to 120° C in five seconds. According to the theoretical curve shown in figure 12, *Z*_2_(*_B_*) never becomes more than about three times greater than it is at 170° C at *any* lower temperature. If this theoretical curve were applicable at such temperatures, a simple calculation shows that it should be easy to quench samples of the polymer that are ~2mm thick into a practically completely amorphous state using the technique indicated. On the contrary, specific volume measurements on clean samples ~2mm thick that had been quenched in ice water from 305° C invariably showed that they possessed x values close to 0.39. This meant that they had completely traversed stage 1 in five seconds or less somewhere between 170° and about 120° C. (It can be shown experimentally that crystallization temperatures lower than about 120° C need not be considered (see below)). These results can only be explained by a log *Z*_2_ value that is at least three decades higher than the theoretical one somewhere between 170° and 120° C. Our conclusion is that there must exist between 120° and 170° C a region of rapid cystallization that is not to be identified as belonging to region B. This conclusion can be shown to hold even if a value of $\Delta H_{p}^{*}+\Delta H_{g}^{*}=0$is used in the analysis of *Z*_2_. Furthermore, the value of *Z*_2_ between 120° and 170° C estimated from the quenching experiments would appear to be too large to be reconciled even with any reasonable visual extrapolation of the *Z*_2_(*_B_*) data actually observed between 170° and 200° C. It may therefore be concluded that the rapid type of nucleation postulated for region C exists in this polymer, and that *T_cc_* = 145 ±25° C. The *T_cc_* value estimated from eq (23) with *σ_s_* = 10.8 erg cm^−2^ and *r*_0_ = 8.4×10^−8^ cm, the latter corresponding to a primary nucleus with seven segments, is about 150° C.

In passing, it should be remarked that it is possible to obtain highly amorphous specimens by quenching sufficiently thin films. For instance, a film 0.12 mm thick gave *x* = 0.10 when heated to 250° C and then plunged into ice water. Samples of this type begin to crystallize slowly when reheated to 100 to 120° C; this suggests that the rapid crystallization characteristic of region C must take place above 120° C. Clean films 0.03 mm thick, when quenched in a similar way, should be less than 1 percent crystalline. The asymptotic specific volumes obtained by quenching various thin films have given additional support to the validity of the expressions for ${\bar{V}}_{l}$ and ${\bar{V}}_{g}$ obtained in a previous investigation [2].

Another point of interest here has to do with two effects that may limit attempts to observe the existence of C-type nucleation in other polymers.

For any reaction that has a negative temperature coefficient, such as the nucléation or growth process in region B, the heat liberated in the reaction will often not be conducted to the external medium with sufficient rapidity, with the result that the reaction will tend to slow down. This will cause the rate of reaction, when plotted as a function of temperature, to possess a broad plateau rather than a sharp peak. This effect is well-known in crystallization mechanisms [26]. In such a case, a considerable portion of the low-temperature part of the rate curve belonging to region B—as well as the transition to region C—will not be directly observable. Because of the remarkably low heat of fusion involved, and the comparatively modest rate of crystallization in region B, this obstacle did not arise in the case of poly(chlorotrifluoroethylene), but it is to be anticipated in other cases. In some polymers, the A→B transition may be obscured by a high rate of crystallization.

Another circumstance that may cause region C to be unobservable is the following. It may happen that the rate of crystallization in region B will have already gone through its maximum, and thus become immeasurably slow prior to the transition to region C. This will tend to occur for polymers where *σ_s_/*Δ*h_f_r*_0_ is large.

No evidence of the existence of region A was found, but this is almost certainly because measurements were not made sufficiently close to *T_m_.* The present work indicates that *T_c_* is above about 205° C, which implies, through eq (17), that *σ_e_* is less than about 0.75 erg cm^−2^. Even if *T_c_* were just at 205° C, crystallization studies of considerable duration would be necessary to obtain the *Z*_2_ values characteristic of region A. Even a search for the onset of region A-type homogeneous nucleation in this polymer is not a particularly inviting prospect because of the long experiments that would be involved.

### 6.5. Summary of Results

It is convenient at this point to summarize the essential results pertaining to the theory of *I*, *G*, and *Z*_2_, and the prediction of the stage 1 isotherms.

In the temperature range 170 to 205° C, which is in region B, the cylindrical critical nucleus has a fixed length *l*_0_ and a variable radius r* = *σ_s_/*Δ*f.* The fundamental physical reason that a fixed length *l*_0_ must be used is that in a higher temperature region, A, the critical length l* decreases approximately as 1*/*Δ*T*, and therefore falls toward a minimum dimension *l*_0_; l* approaches *l*_0_ at a relatively high temperature, *T_c_*, because of the smallness of *σ_e_* in the expression l* = 4<*σ_e_*/Δ*f*. The existence of an irreducible segment length *l*_0_ is demanded by the discrete character of the links that comprise the linear polymer chain. Region B commences when l* reaches *l*_0_, and comes to an end at a much lower temperature, *T_cc_*, where the critical radius of the nucleus, r* = *σ_s_/*Δ*f*, falls to a value of molecular dimensions, *r*_0_. The wide range of temperature encompassed by region B is a result of the fact that *σ_s_* is considerably larger than *σ_e_.*

The rate of injection of the nuclei into the supercooled liquid phase in region B is given by

$$I_{B}=I_{0}e^{-\Delta H_{p}^{*}/RT}e^{-\pi T_{m}^{2}l_{0}\sigma_{s}^{2}/\Delta h_{f}T\Delta TkT}.$$ (36)

The homogeneous nucleus arises spontaneously at random times and positions in the supercooled liquid as a result of alinement of segments of different polymer molecules. The last term in eq (36), i.e., the nucleation term, dominates the temperature dependence of *I* in the experimental range studied. The term $\exp(-\Delta H_{p}^{*}/RT)$ controls the short-range diffusion process at the supercooled- liquid—nucleus interface. The critical nucleus grows in a radial manner up to a mean radius $\bar{r}$ in a time that is very short compared to its own birth time. Here it stops growing radially due to impingements or volume strain. Thus, the actual homogeneously induced nucleation center has a radius $\bar{r}$ and a length *l*_0_, where $\bar{r}>l_{0}$. It thus resembles a platelet or flat cylinder, where the long axes of the polymer molecules are normal to the radius. An equation differing from (36) because it is based on a nucleus with unrestricted *l* will apply near *T_m_.*

The homogeneously injected nucleation center grows along the *l* direction, i.e., the platelet thickens. The lineal growth process by which the platelet thickens is controlled by a secondary or growth nucleus of radius ρ* = *σ_s_*/Δ*f* and length λ_0_. (The quantity λ_0_ is numerically equal to *l*_0_.) The lineal growth rate is given by

$$G=G_{0}e^{-\Delta H_{g}^{*}/RT}e^{-\pi Tm^{2}\lambda_{0}\sigma_{s}^{2}/\Delta h_{f}T\Delta TkT}.$$ (37)

The temperature dependence of *G* is controlled by the nucleation term in the experimentally accessible range, so that *G*, just like *I*, has a negative temperature coefficient. The lineal growth process eventually stops, or at least slows down markedly, as a result of impingements at the pseudoequilibrium degree of crystallinity, *x_m_.*

The overall crystallization rate is proportional to the area of the growing crystal face, the rate of injection of nuclei, and the lineal growth rate. The quantity *Z*_2_ in the expression *x*′ = *Z*_2_*t*^2^, which is that appropriate to one-dimensional growth of objects born sporadically in time, is thus proportional to $\pi {\bar{r}}^{2}\;IG$. Hence

$$Z_{2(B)}=Z_{0}e^{-(\Delta H_{g}^{*}+\Delta H_{p}^{*})/RT}e^{-\pi T_{m}^{2}(l_{0}+\lambda_{0})\sigma_{s}^{2}/\Delta h_{f}T\Delta TkT}$$ (38)

*Z*_2_ is directly measurable from the *n* = 2 isotherms, so that (*l*_0_+λ_0_)*σ_s_*^2^ can be evaluated. The nucleation exponent in *G* can be determined from the *n* = 1 seed crystal isotherms, and λ_0_*σ_s_*^2^ also evaluated directly. This latter value proves to be very nearly the same as the λ_0_*σ_s_*^2^ value calculated from the *Z*_2_ data using *l*_0_. = λ_0_. Thus, the nucleation exponents in eqs (36 to 38) are shown to be in the ratio 1:1:2, as required by the theory. This provides a strong argument for the validity of the present approach.

The original *n* = 2 isotherm data up to *x* = 0.3 can be reproduced extremely well by inserting values of the appropriate parameters gathered together in table 5 into eq (38), calculating *Z*_2_ as a function of temperature, and then calculating the isotherms for each temperature using the expression *x* = [1 – exp (−*Z*_2_*t*^2^)], i.e., eq (4) with *x_w_* = 1. Furthermore, the observed temperature dependence of *G* can be reproduced by using the appropriate input data with eq (37).

## 7. Bulk Crystallization and Spherulitic Growth

The principal objective of this section is to dispel any notion that the intrinsic bulk crystallization process in poly(chlorotrifluoroethylene) is of a spherulitic character, or in any other manner involves spherical growth, in the temperature range studied. The remarks given below concerning the absence of indigenous spherical growth in this polymer thus refer specifically to region C, and region B up to ~205° C. The possibility that intrinsic spherical growth may develop in region A will also be dealt with briefly.

It was demonstrated in section 3 that when poly(chlorotrifluoroethylene) is crystallized subsequent to strong superheating, the bulk crystallization accurately follows an *n* = 2 law. This value of *n* is, of course, inconsistent with spherical growth generally; the latter leads to *n* = 3 for objects born at *t* = 0, and *n* = 4 for objects born sporadically in time. However, for low degrees of superheating, the isotherms are in fact quite closely representable by an *n* = 3 law. An example is the *T*_1_ = 245° C isotherm shown in figure 1. It must be clearly understood that the *n* = 3 behavior in such a case is beyond doubt connected with the presence of heterogeneities. This is shown by the facts that (a) as is evident in figure 1, increased superheating leads to slower crystallization at a given growth temperature which must result from the destruction of embryos in fissures in the heterogeneities and (b) that as these embryos are progressively destroyed by increased superheating, the isotherms go from *n* = 3 asymptotically toward *n* = 2. The only meaning that we can attach to the above is that spherical (*n* = 3) growth originates only at embryos retained on heterogeneities. The above is consistent with the idea that the *n* = 2 isotherm is an intrinsic property of the polymer.

Although the evidence cited above is quite sufficient to completely eliminate all forms of spherical growth (including the spherulitic) as the intrinsic crystallization mechanism in poly(chlorotrifluoroethylene), it is still of interest to prove this directly for the specific case of spherulites in order to further corroborate the point of view expressed here.

It has been demonstrated by Price [9] that spherulites in this material must originate at heterogeneities. In successive runs, virtually all of the spherulites reappeared at the same site. Further, Price demonstrated that the number of spherulites present in a sample depended strongly on the degree of superheating, the number decreasing sharply with increased superheating. This shows conclusively that the spherulites were born specifically at embryos in fissures or pores in the heterogeneities, as Price points out. All of the above experimental results of Price have been confirmed for the spherulites that appear in the specimens used in this investigation [10]. In particular, most of them are bom near *t* = 0 even in strongly superheated specimens. If spherulites born near *t* = 0 were the main seat of the crystallization in the system the bulk crystallization would follow an *n* = 3 law, since the radial growth rate is constant with time.

The fact that the spherulites originate at heterogeneities, and decrease in number with increased superheating, is completely in accord with the remarks given above concerning the absence of intrinsic spherical growth generally. As the number of spherulites is decreased through the agency of increased superheating, the *n* values characterizing the corresponding bulk crystallization isotherms go from *n* = 3 (or *n* = 3^+^ if low growth temperatures are considered) down to *n* = 2. The value *n* = 2 must therefore correspond to the case where the effect of spherulitic growth is nil.

Further evidence showing that spherulites cannot be at the root of the intrinsic bulk crystallization in the *n* = 2 runs can be given. As indicated in section 2.1, the specimens for which the reproducible isotherms were obtained actually still contained from 10 to 30 spherulites/mm^3^ even after superheating to 305° C. While the corresponding isotherms were *n* = 2, which eliminates the possibility of the dominance of spherical growth generally, it was still considered of interest to compare the volume fraction of spherulites with the known (bulk) degree of crystallinity known to be present in such samples.

It is possible to demonstrate that the volume fraction of spherulites in the relatively early part of the crystallization of the *n* = 2 specimens lags behind the volume fraction actually known to be crystallized *even when the spherulites are reckoned as solid objects* [10].^12^ Even a casual microscopic examination with polarized light shows that the spherulites in poly(chlorotrifluoroethylene) are far from solidly crystalline. This is especially true for spherulites that are formed at high growth temperatures. In the latter case, the spherulites are almost transparent, and the characteristic maltese cross barely discernible, despite the fact that the spherulite boundaries are clearly visible. Thus, the volume fraction of truly spherulitic material must lag far behind the actual volume fraction crystallized. On the basis of the above information alone, it can be concluded that spherulites cannot possibly be the seat of the crystallization in the *n* = 2 specimens.

Another point worth mentioning is that it is possible to prepare highly crystalline samples (*x*>0.60) of poly(chlorotrifluoroethylene) that contain no spherulites whatever, and are optically clear [2]. Spherulites never appear in this polymer below ~150° C, even though the necessary heterogeneities are present [9]. This may be attributed to the onset of region C-type homogeneous nucleation, which simply overwhelms the spherulitic growth. The optically clear and highly crystalline samples just mentioned were made by first “quenching” them from 305° C to 0° C, which for samples of the thickness used amounts to nucleation and growth up to stage 2 in region C, and then continuing the crystallization at a higher temperature in order to take advantage of the increased velocity of stage 2 in that region (see figure 2).

*It may be concluded that neither spherical nor spherulitic growth is dominant in the intrinsic bulk crystallization process in poly*(*chlorotrifluoroethylene*) *below ca 205° C. The evidence points without exception to the fact that the vast majority of spherulites found in this substance in the temperature range mentioned are to be regarded as artifacts*, *directly traceable to heterogeneities, that can be eliminated to a sufficient degree by appropriate superheating and selection of samples. While the few spherulites that are present appear at first sight to be so obviously the seat of the crystallization in the n* = *2 specimens*, *careful consideration of the facts proves this to be incorrect.*

The crystallinity in the *n* = 2 specimens consists of vast numbers of tiny crystallites that are dispersed throughout the polymer. These crystallites must possess a distribution of sizes, but the majority of them are evidently too small (less than *ca*.1000 A) and randomly oriented to be detected easily with a polarizing microscope. The weakly translucent appearance of many of the *n* = 2 samples is mostly a result of stray spherulites. *The intrinsic bulk crystallization in poly*(*chlorotrifluoroethylene*) *in region C and region B up to ~205*° *C resides in the essentially optically structureless background.* The overall gray cast with a somewhat grainy texture that is often seen in samples of poly(chlorotrifluoroethylene) with a polarizing microscope is due to surface nucleation [11], and should not be confused with the ultra-fine-grained bulk crystallization which does not scatter visible light to a marked extent. The ultra-fine-grained crystallization mentioned here is evidently of the same general type that is frequently encountered in X-ray investigations on other semicrystalline polymers.

The crystallites in the *n* = 1 seeded specimens are larger than those in the *n* = 2 specimens, since the former samples have a chalky-white rather than weakly translucent appearance. This occurs because the largest centers are selected for regrowth for samples seeded in the manner described.

The possibility that intrinsic spherical growth may develop in region A will now be considered. Although it was not feasible to determine either *n* or the rate of crystallization in this region for poly(chlorotrifluoroethylene), the remarks below are nevertheless appropriate in view of the fact that a plausible case can be made for homogeneously- initiated spherulitic growth near the melting point of certain polymers (see for example the work of McIntyre [34], and McIntyre and Flory [35]). It is interesting to note that Flory and McIntyre achieved homogeneous initiation only for filtered samples that had been superheated prior to crystallization.

It will be observed from a comparison of eqs (16), (22), and (27) that the ratio of the rate of injection to the rate of growth will be much smaller in region A than in region B. When this fact is considered together with the nature of impingements, it is seen that the average (grown) crystallite size will be considerably larger in region A than it is in region B. The authors consider it unlikely, owing to the effect of various possible imperfections, that a polymer crystal will grow to really large size along the simple pattern established for small crystallites. If somewhere in region A the homogeneous nuclei are formed sufficiently far apart, the growth in either the *r* or *l* direction should often develop as the result of some type of imperfection a number of branch points prior to impingement, with the result that the growth will become three-dimensional. In this event, the isotherm would go progressively from *n* = 2 to *n* = 4, the latter corresponding to threedimensional (possibly spherulitic) growth of objects born sporadically in time. No detailed consideration will be given to the possible origin of the branching mechanism, but the following may be mentioned: (a) Faults due to chain branching or large chain ends; (b) spiral dislocations; (c) secondary nucleation at preferred sites or orientations; (d) evasion of certain types of impingements; and (e) preferential primary nucleation at certain sites [34, 35].

It is conceivable that the tendency toward threedimensional growth could begin somewhere in region B, especially if the branches developed early in the growth of a crystallite. The point here is that the change-over from region B to region A-type homogeneous nucleation will not necessarily be marked closely by the change of *n* from 2 to 4, or by the onset of intrinsic spherulitic growth as observed by microscopic examination. This will be particularly true if chain ends are the cause of the branching mechanism leading to three-dimensional growth, for here the inception of such growth will depend on molecular weight.

Our conclusion is that intrinsic spherical (and possibly spherulitic) growth with *n* = 4 is neither theoretically forbidden by the present scheme, nor even unlikely, especially at temperatures sufficiently near *T_m_.* Another point that cannot be emphasized too much is the probable role of impingements in determining the mode of growth of polymeric crystals.

## 8. Concluding Remarks

Now that the experimental and theoretical discussions of *I*, *G*, and *Z*_2_ have been given, and the role of spherulitic or three-dimensional growth placed to its proper position for poly(chlorotrifluoroethylene), it is convenient to take up two questions that deserve consideration. The first of these has to do with the extent to which it may be believed that an intrinsic property of the polymer was studied. The second deals with the possible application of the theory to other systems.

### 8.1. Evidence for Homogeneous Nucleation

It was shown by a comparison of the *n* = 1 and *n* = 2 results that the primary nuclei must have been born at later and later dates. Such behavior is a characteristic of true homogeneous nucleation. It is also a characteristic of pseudohomogeneous nucleation, where growth centers may be born at essentially later and later times, at least early in the transformation, on flat surfaces that are only slightly wettable by the crystalline phase. (Nonwettable heterogeneities are inactive as nucleation centers.) Now it is certain from the superheating studies shown in figure 1 that some heterogeneities are present even in the best specimens, and that they are wettable to a certain extent. Therefore, it is necessary to consider the possibility that the nuclei that were born later and later in time in the *n* = 2 runs which were obtained subsequent to strong superheating were of pseudohomogeneous rather than homogeneous origin.

Strong evidence that points directly to a preponderance of homogeneous initiation in the *n* = 2 runs is afforded by the fact that the nucleation exponents in eqs (36 to 38) are found experimentally to be very nearly in the ratio 1:1:2. This means that the *σ_s_* value in the expressions for *I, G*, and *Z*_2_ must refer to the same quantity in all cases. If the *σ_s_* value in the expression for *I* was in fact an interfacial free energy of the system crystal+foreign substrate, (i.e., of the form *σ_s_f*(cos *θ*), where *θ* is the contact angle) it could not, in view of the proven wettability of the heterogeneities, be so nearly the same as the *σ_s_* value found in the expression for *G*, the latter obviously applying to the supercooled- liquid—crystallite interface. The *σ_s_* values determined independently from the *Z*_2_ and *G* data are within 5 percent of each other. The small difference, which is in the wrong direction to be explained by a cos *θ* term in the expression for *I*, is ascribable to the experimental error in *G_i_/G_j_.*

Another point favoring homogeneous nucleation in the *n* = 2 runs is that the *σ_s_* value obtained is in consonance with the observed surface free energies of chemically similar but low molecular weight materials [33] where the experimental conditions were clearly those conducive solely to homogeneous nucleation. However, this argument is by no means conclusive when considered by itself, especially in view of the fact that *σ_s_* depends on the estimate of *l*_0_.

A simple calculation based on the X-ray results, and the fact that the crystallites do not scatter light to any great extent, show that there must be at least 10^17^ of them in a cm^3^ of polymer. Such a result is easily understood in terms of the homogeneous initiation and secondary growth scheme proposed in this paper: the ratio of the rate of injection to the rate of growth is such that a very fine-grained crystallinity is expected at the point of massive impingement. In order to revive the idea that the primary nucleation is actually mainly pseudohomogeneous, it would be necessary to postulate that a population well in excess of 10^17^ thermally stable and very weakly wettable foreign bodies existed in each cm^3^ of polymer. These foreign bodies would have to be very weakly wettable in order to lead to essentially sporadic initiation, and in order to explain the fact that *σ_s_* was the same when calculated from *Z*_2_ and *G.* The spherulite-producing heterogeneities are not only far too small in number, but are also much too wettable to be identified with any hierarchy of foreign particles capable of leading to pseudohomogeneous initiation of ~10^17^ crystallites per cm^3^. While we do not completely discount the possibility that a set of >10^17^ barely wettable and thermally stable centers for pseudohomogeneous nucleation may exist in each cm^3^ of polymer, the existence of such a prodigious number of them with such special physical properties appears to us rather unlikely. The existence of the ultra-fine-grained crystallinity seems more readily explained in terms of a homogeneous initiation mechanism.

An effect that might be expected in the case of pseudohomogeneous nucleation would be a shift of *n* with growth temperature. This would occur as a result of a decrease of wettability of the heterogeneities with falling temperature. The excellent superposability obtained with the *n* = 2 isotherms attests to the fact that *n* does not shift appreciably with temperature. This is also shown by the *n* values listed in table 1 ; within experimental error, there is no persistent trend in the data.

When considered together, the arguments cited above provide substantial reason for the belief that the injection of primary nuclei in the *n* = 2 runs was mostly a result of true homogeneous nucleation. It is emphasized that this holds only for those specimens that were carefully selected so that they contained a minimum number of heterogeneities, and which were strongly superheated prior to crystallization.

The samples of poly(chlorotrifluoroethylene) commonly available usually contain so many heterogeneities that even strong superheating is relatively ineffective, with the result that the intrinsic properties of the polymer remain hidden in bulk crystallization studies on such material. This type of material is generally highly spherulitic when crystallized, and follows an *n* ≅ 3 bulk crystallization law.

### 8.2. Application to Other Systems

It seems appropriate to mention certain ramifications of the present theory that may have an influence on any attempted application to other systems.

One likely deviation from the present analysis will be caused by variations in the mode of growth. This will have an effect on *n*, and the analysis of the growth rate. It is emphasized again that the value *n* = 2, which prevailed in the case of poly(chlorotrifluoroethylene), is intimately related to both the nature of impingements and to the relative rate of nucleation and growth. The impingements stopped the rapid radial growth of the thin disks at $\bar{r}$ before any serious branching took place. The relatively high rate of primary nucleation led to early edgewise impingement, and the amount of crystallization involved was negligible because of the thinness of the disks. As a consequence, the observable growth process was one-dimensional. Such a set of circumstances, while possibly common, is certainly not to be expected in all crystallizable linear polymers, or even in a given polymer over an extremely wide range of temperature.

It has already been pointed out how intrinsic spherical growth leading to an *n* = 4 bulk crystallization isotherm might develop in region A, or perhaps in the upper part of region B if the primary nuclei were formed sufficiently far apart. It remains to be mentioned how a bulk crystallization isotherm with *n* = 3 could prevail under homogeneous nucleation conditions. If the radial growth process described by *G_r_* were of measurable magnitude, and the thickening of the platelets along the *l* direction relatively much slower, then an *n* = 3 isotherm due to radial growth of disks born sporadically in time would result up to the point of impingement. Such a condition may be best fulfilled in region A where the primary nucleus is rather large, and the rate of injection low. Lengthwise growth of the nuclei may tend to be “poisoned” by the advent of end-groups that are too large to be accommodated even in a disordered crystal. The point here is that *n* = 2, *n* = 3, or *n* = 4 intrinsic bulk crystallization may arise depending on the mode of growth. In turn, the mode of growth may depend on the nature of impingements and the details of chain structure.

A drastic reduction in the lengthwise growth rate may occur in polymers that are partially atactic, and partially iso tactic (or syndiotactic), and possess large side groups. The primary nuclei would tend to form from the isotactic portions of the chain. Then, as the lengthwise growth proceeded, atactic portions would be encountered. If the lattice mismatch was bad, the lengthwise growth would probably be greatly slowed down. The maximum degree of crystallinity attainable would also be reduced. The lattice mismatch would be much less serious if the side groups were small. There is no evidence of a diminution of the growth rate in poly(chlorotrifluoroethylene), or of an unusually low limit on the degree of crystallinity. It is therefore reasonable to suppose that the polymer supplied was either largely isotactic or syndiotactic, or that the Cl side group was not sufficiently different in size from the others to prevent the formation of a (rotationally disordered) crystal from the atactic polymer. The former possibility seems the most likely.

In region C, the embryos transported from the superheated state will in some cases become nuclei stable at *t* = 0. This will cause the *n* value to be lower than it would be in the case of nuclei born sporadically in time. Crystallization studies are sometimes made by reheating thin quenched films to the growth temperature. It would appear likely that such specimens would contain predestined nuclei, and thus behave, at least in part, like those that had been deliberately seeded. This factor renders uncertain the interpretation of some of the low-temperature rate of crystallization data in the literature.

The use of seed crystals derived from a previous crystallization carried out under conditions conducive to homogeneous nucleation is an important part of the procedure used in the present paper. In general, the seed-crystal isotherm should have an *n* value lower by unity than the homogeneously-initiated isotherm. The reader is cautioned not to use foreign particles as the “seeding” agent, since these will frequently induce a spherulitic mode of growth in a temperature range where the intrinsic mechanism may not be spherulitic.

The statement, or at any rate the implication, is sometimes found in the literature that, in careful work, the intrinsic mode of growth is always found to be spherulitic. The present work would suggest on both theoretical and experimental grounds that no such general concession to the primacy of intrinsic “*n* = 4” or spherical growth at all temperatures be made. As noted previously, homogeneously induced spherulitic growth should and evidently does exist, [34, 35]. However, the present work provides what in our view is an equally credible demonstration of homogeneously induced one-dimensional growth leading to *n* = 2 bulk crystallization isotherms. The growth mechanism in this case is in fact among the simplest and most straightforward imaginable. We would also emphasize again the possibility that homogeneously induced disk-like growth leading to *n* = 3 isotherms may arise in certain polymers.

In other presentations, a single expression, usually of the general form of *I_A_*, is often used to describe the rate of primary nucleation in bulk polymers at all temperatures below *T_m_.* It would be extremely curious if *I_A_* applied at all temperatures for all polymers. The expression for *I_A_* is derived on the assumption that *r* and *l* are unrestricted by any “minimum” molecular dimensions. Remembering that the size of the nuclei in region A depends on certain surface free energies and the temperature, the latter in such a way as to cause the nuclei to rapidly diminish in size with an increase of Δ*T*, and recalling that polymer chains are commonly regarded as having a segmental character, it would be surprising indeed if at some temperature a restriction from *l*, and later *r*, did not require consideration. In any event, we have shown that effects resulting to this general type of restriction enter in the case of poly(chlorotrifluoroethylene).

It would be of considerable interest to carry out precise experiments designed to reveal the A→B and B→C transitions. In carrying out such studies, it should be borne in mind that a rapid nucleation- controlled growth mechanism may tend to obscure details of the primary nucleation, especially in the case of two- or three-dimensional growth. Thus, for two-dimensional growth, one might have $Z_{3}\propto I_{A}G_{r}^{2}$ or $Z_{3}\propto I_{B}G_{r}^{2}$ where *G*_r_≫*G;* here it would be quite difficult to see the A→B and B→C transitions experimentally. As one would expect, studies on nucleation and growth mechanisms in nonpolymeric systems reveal no A→B transitions, and the primary nucleation follows a Δ*T*^−2^ law.

The foregoing covers some of the more obvious possible extensions of the present type of analysis, and highlights a few of the difficulties that may be encountered in any attempted application. Even with this, the presentation is incomplete. For instance, the theory for a bulk homopolymer where *σ_e_* is comparable to *σ_s_* has not been pursued, nor have the interesting details of the A→B and B→C transitions been fully delineated. Nevertheless, the theory given does seem to illuminate certain aspects of the intrinsic bulk crystallization phenomenon in polymers to the limited extent to which it is now known.

## Figures and Tables

**Table 1:** Values of n and x_w_ for polymer crystallized after strong superheating (homogeneous or pseudohomogeneous nucleation)

Initial temperature, *T*_1_	Crystallization temperature, *T*_2_	Value of *n*		*x_w_*	Maximum experimental *x* value that is approximately fitted by eq (4) with *n* =2 and *x_w_*=1	Pseudoequilibrium degree of crystallinity, *x_m_*
		eq (5) with *x_w_*= 1 assumed	eq (5) with *x_w_*=½ assumed			
						
° *C*	° *C*					
305	199.9	a2.0	a 2.1	………	…………………………………………………………………………………	……………………………………………
305	195.8	1.86	1.96	1.	0.50	0.60
305	192.5	1.84	1.94	.75	.50	.57
305	188.4	1.98	2.09	1.	.45	.53
305	184.4	2.01	2.13	1.	.40	.52
305	180.4	2.10	2.22	1.	.40	.50
285	188.4	2.06	2.19	1.	.45	…………
		*n*=**1.97**	*n*=2.09	~**1**		

a For this run, there were too few points taken in the region *x*=0.05 to *x*=0.20 to permit *n* to be calculated as precisely as in the other cases.

**Table 2:** Values of n and x_w_ for polymer nucleated with seed crystals^a^

Initial temperature, *T*_1_	Growth temperture *T*_2_	Value of *n*		*x_w_*	Maximum experimental *x* value that is approximately fitted by eq (4) with *n*=1 and *x_w_*=0.55	Pseudoequilibrium degree of crystallinity, *x_m_*
		eq (5) with *x_w_* = 1 assumed	cq (5) with *x_w_* = ½ assumed			
						
° C	° C					
216.2	200.0	0.98	1.04	0.55	0.40	~0.45
215.6	200.0	1.02	1.08	.55	.40	~.45
215.6	196.2	.87	.92	.55	.40	~.45
		*n* = 0.96	*n* = **1.01**	**.55**		

a Previous history prior to heating to *T*_1_ value shown in table: *n* = 2 run with *T*_1_ = 305° and *T*_2_ = 188° C that was carried into stage 2.

**Table 3:** Z_2_ as a function of temperature calculated from n = 2 isotherms^a^

Crystallization temperature, *T*_2_	*Z*_2_	log *Z*_2_
		
° *C*	*sec*^−2^	
170.0	b(4.4×10^−6^)	b(−5.36)
180.4	4.80×10^−7^	−6.319
184.4	1.25×10^−7^	−6.903
188.4	2.33×10^−8^	−7.633
192.5	3.36×10^−9^	−8.474
195.8	3.44×10^−10^	−9.463
199.9	6.76×10^−12^	−11.170

a For *T*_1_ = 305° C.

b Approximate value.

**Table 4:** Relative values of G for T_1_ = 215.6° C from n = 1 seed crystal isotherms^a^

Growth temperature, *T*_2_	Time required to reach *x* = 0.15	Ratio of growth rates, *G_i_/G_j_*
		
*° C *	*sec*	
$I\{\begin{array}{l} 196.2\\ 203.9 \end{array}$	9.30×10^2^	$\begin{array}{ll} \} & \frac{G_{196.2{}^{\circ}}}{G_{203.9{}^{\circ}}}=74 \end{array}$
	6.90×10^4^	
$\text{II}\{\begin{array}{l} 191.5\\ 205.2 \end{array}$	2.43×10^2^	$\begin{array}{ll} \} & \frac{G_{191.5{}^{\circ}}}{G_{205.2{}^{\circ}}}=1570 \end{array}$
	3.81×10^5^	

a Previous history prior to heating to 215.6° C: *n* = 2 run carried well into stage 2 with *T*_1_ = 305°, *T*_2_ = 188° C for pair I, and *T*_1_ = 305°, *T*_2_ = 180° C for pair II.

**Table 5:** Input data and summary of results

Quantity	Value and units	Remarks

Input data		
*T_m_*	494.2° K	From melting point studies [12]
${\bar{V}}_{e}$	0.473 cm^3^g^−1^	Mean value for temperature range 170° to 200° C [2]
${\bar{V}}_{1}$	0.522 cm^3^g^−1^	Do.
Δ*h_f_*	9.10×10^8^ erg cm^−3^	Calculated from Bueche’s value Δ*H_f_*=10.3 cal g^−1^ [28], and ${\bar{V}}_{e}$
$\Delta H_{p}^{*}+\Delta H$	32,000 cal mole^−1^	From dielectric data [27]
$\Delta H_{g}^{*}$	16,000 cal mole^−1^	Do.
*l*_0_,*λ*_0_	10×10^−8^ cm	Based on assumption crystallizing segment contains 4 monomer units
*M*	4×116.5=466 g mole^−1^	Do.
$\bar{r}$	~100×10^−8^ cm	From X-ray data [29]^a^
*d*	5.6×10^−8^ cm	From X-ray data [30, 31]
*r*_0_	8.4×10^−8^ cm	For nucleus containing seven segments

Results		
(*l*_0_*+λ*_0_)*σ*_8_^2^	$\{\begin{array}{c} 1.96\times 10^{-5}{\text{erg}}^{2}{\text{cm}}^{-3}\\ 2.31\times 10^{-5}{\text{erg}}^{2}{\text{cm}}^{-3} \end{array}$	From *Z*_2_ data with $\Delta H_{p}^{*}+\Delta H_{g}^{*}=0$ Best value from *Z*_2_ data $\left(\Delta H_{p}^{*}+\Delta H_{g}^{*}=32,000\;{\text{cal}\;\text{mole}}^{-1}\right)$
*l*_0_*σ*_8_^2^ λ_0_*σ*_8_^2^	}1.15×10^−5^ erg^2^ cm^−3^	Best values from *Z*_2_ data calculated as (*l*_0_+*λ*_0_)*σ*_8_^2^/2
*λ*_0_*σ*_8_^2^	1.05×10^−5^ erg^2^ cm^−3^	From *G* data
log *Z*_0_	16.47	Best value from *Z*_2_ data
$\Delta S_{p}^{*}+\Delta S_{g}^{*}$	−56.8 cal mole^−1^ deg^−1^	Do.
*σ*_8_	10.8 erg cm^−2^	Do.
*σ*/Δ*H_f_*	0.21	…………….
_r*_	$\{\begin{array}{c} 12.8\times 10^{-8}\text{cm}\;\text{at}\;170{}^{\circ}C\\ 29\times 10^{-8}\text{at}\;200{}^{\circ}C \end{array}$	For *σ*_8_=10.8 erg cm^−2^
*T_c_*	>205°C	…………….
*σ_e_*	<0.75 erg cm^−2^	Based on *T_c_*
*T_cc_*	$\left\{ \begin{array}{l} 145\pm 25{}^{\circ}C\\ \sim 150{}^{\circ}C \end{array}\right.$	From quenching studies For *σ*_8_=10.8 erg cm^−2^, *r*_0_=8.4×10^−8^ cm

a The quantity $\bar{r}$ is about 50A in stage 1, and about 200 A in stage 2. All val lies of $\bar{r}$ quoted in the paper were calculated on assumption X-ray line broadening was a result of crystallite size effects.

## Footnotes

1 Figures in brackets indicate the literature references at the end of this paper.

2 A total absence of superheating effects would mean that the corresponding crystallization was of homogeneous origin. This represents an experimentally ideal situation not easily achieved. However, the observation of only a slight displacement at constant *T*_2_ toward slower crystallization with increasing *T*_1_ at relatively low degrees of superheating does not necessarily imply that such isotherms are of practically homogeneous origin. This may be seen by the following argument. Suppose the crack-size distribution in the heterogeneities is gaussian, with a most probable size $\bar{x}$. At low degrees of superheating only the very few large embryos will be melted out, and the isotherms will shift but little with increasing *T*_1_, despite the fact the crystallization is heterogeneous. The maximum shift will correspond to higher *T*_1_ values where embryos in cracks of just the size $\bar{x}$ are melted out. At still higher *T*_1_ values, the isotherms will converge again as shown in figure 1 as the last of the small embryos is destroyed by superheating. Thus, even if only a small effect of superheating is found, it is important to show, as in the present case, that the effect of further superheating causes *convergent* shifts before it can be stated that a homogeneous or pseudo homogeneous condition of crystallization has been approached. A *divergent* shift would be a certain sign that the crystallization was mostly initiated at *t*=0.

3 Equation (4) is not quoted directly in the papers cited, but is readily deduced from the treatment given.

4 An incidental point here is that *n* = 4 is in itself not a complete proof of homogeneous nucleation, but only of essentially sporadic initiation. Such an effect could arise from either homogeneous or pseudohomogeneous nucleation. In this sense, an *n* = 4 isotherm is somewhat less definite with regard to the type of nucleation that is implied than an *n* = 1 isotherm, the latter practically always clearly indicating heterogeneous nucleation. (Here we regard both foreign bodies and seed crystals as “heterogeneous” nuclei). An *n* = 1 isotherm could conceivably arise for a substance that “crystallized” strictly by continuous homogeneous injection of nuclei that did not grow, but this contingency need not be seriously considered in the present case, since there is ample evidence showing that the primary nuclei grow.

5 The fact that *T_m_*′ is less than *T_m_* for polymer crystallized at temperatures below *T_m_* is due to the small size of the crystals in such samples. The specimens will contain crystallites with a distribution of sizes, the larger ones melting out at higher temperatures. Some of these will be retained above the nominal value of *T_m_'.* The number of seed crystals in a sample will tend to depend rather strongly on *T*_1_ in the range between *T_m_'* and *T_m_.*

6 Definitions of *σ_s_, σ_e_*, and *ϵ* are as follows. Let *W*_1_ be the work required to isothermally draw a segment of length *l*_0_ from the center of the crystal to the lateral surface, and let *d* be the lattice side-spacing. Then *σ_s_* = *W*_1_/*l*_0_*d*. If *W*_2_ is the work required to move a segment from the center to the middle of the end of nucleus, then *σ_e_* = *W*_2_*/d*^2^. Define *W*_3_ as the work required to isothermally draw a segment from the center of the crystal to the site of the edge phase. Then *ϵ*, as treated here, is approximately (*W*_3_–*W*_1_–*W*_2_*)/d.* (This particular expression holds exactly only for a parallelipiped, but is sufficient to indicate the meaning of *ϵ*). *W*_3_ and *W*_1_ are both large, and *W*_2_ is small, so that *ϵ* will be a very small residual. A definition of the edge free energy ℰ (as opposed to the *residual* edge free energy *ϵ*) involving only *W*_3_ could be given, but its use would have required a re-definition of the lateral surface area, as well as other complications, and we have chosen not to use it.

7 The authors are indebted to Dr. D. Turnbull and Dr. F. P. Price of the General Electric Research Laboratory for suggesting the use of this model in the present application, and for pointing out certain features of the derivation given for Δ*ϕ^*^.*

8 The view has been expressed that primary polymer nuclei will always have a free energy of formation of 8*πT_m_*^2^*γ*^2^*γ_e_/*Δ*h_u_*^2^Δ*T*^2^ (analogous to our 8*πσ_s_*^2^*σ_e_/*Δ*f*^2^ with the approximate form Δ*f*≅Δ*h_f_*Δ*T/T_m_*), and this has led to the assertion that a primary polymer nucleus with fixed length is of an extremely arbitrary character. The origin of this view appears to be the belief that expressions of the form of eq (21) can be derived only on the basis *σ_e_≡*0. A value *σ_e_*≡0 would tend to imply that the parameter *γ_e_* = *RT* In *D* in Flory’s theory [15, 21] was identical to zero, and this is generally conceded to be impossible. One of the physical reasons for believing *γ_e_*>0 is very similar to the argument we have given for *σ_e_*>0. In any event, the derivation of eq (21), which involves a nucleus of fixed length, in no way implies that *σ_e_* is zero, but only that it is small compared to *l*_0_Δ*f*. Thus, neither eqs (20) or (21) stand in contradiction to Flory’s theory in this respect, and the concept that a primary nucleus may have fixed length in the appropriate temperature interval is in no way arbitrary. We have left out of consideration the possibility that *γ_e_*, which is the free energy of formation of the end of an equilibrium-sized crystallite with a cross-section of *ρ* chains, may not be precisely the same as the free energy of formation of the end of a primary nucleus of the same cross section.

9 Strictly speaking, the ${\bar{V}}_{0}$ used by Doolittle is called the “occupied volume”, and is defined as the limiting volume to which a real liquid will contract if it were to continue to behave as a nonassociated liquid without change of phase all the way to 0° K.

10 The constants in the Williams, Landel, and Ferry equation were derived empirically on the assumption that *E** = 0. An expression that applied even above *T*_g_+100° might be obtained by using other values of *E*.* This would probably require a slight revision of the constants in the Williams, Landel and Ferry expression for *η/ηT_g_.* The authors gratefully acknowledge a private communication from Professor Ferry on this subject.

11 An equilibrium melting temperature experiment of the conventional type, where very slow heating was used, provided further evidence for believing that *T_m_* is within the range indicated.

12 This finding eliminates from consideration spherulites born at *t* = 0 whose crystal content develops as *n* = 2, but whose optically apparent volume develops as *n* = 3. Spherulites of this type, where the crystal density attenuates with growth, arc also improbable for the reason that in specimens where spherulites born at *t* = 0 are extremely numerous, the bulk crystallization follows an *n* = 3 law.

## 9. Appendix

### 9.1. Derivation of Equation (4)

From a formal standpoint, the derivation given below is based on the treatment due to Mandelkern [5], and Mandelkern, Quinn, and Flory [6]. The principal reasons for repeating this derivation here are (a) to clarify the meaning of *x_w_* (their *x_w_* or *λ_w_*), and to indicate why it is actually not precisely invariant with *x*, (b) to show how their approach may be applied directly to the particular crystallization variable, *x*, used in the body of the present paper, and (c) to indicate the theoretical values assumed by *x_w_* in several situations of interest. The derivation is intended to apply to homopolymers that can eventually achieve a high degree of crystallinity.

Consider a polymer specimen of total mass *M*_0_. Let $dM_{c}^{\prime }$ be the increment of mass transformed into the crystalline state in a time *dt* on a *free growth basis*, so that $M_{c}^{\prime }$ is the mass at time *t* on the same basis. Take *dM_c_* to be the *actual* mass transformed in an interval *dt*, so that *M_c_* is the actual mass transformed up to *t.* The fraction *M_c_/M*_0_ is *x*, and *dM_c_*/*M*_0_ is *dx.* It is assumed that *M_c_* is less than $M_{c}^{\prime }$ at any given time owing to various retardations to the growth, and the depletion of crystallizable material. The mass fraction transformed on a free growth basis, *x′*, is $M_{c}^{\prime }/M_{b}$. This quantity is called the “free bulk growth rate” in the text.

Define a retardation parameter *x_w_*, where *x_w_*>0, as the *mean value of the apparent limiting degree of crystallinity in stage 1.* This definition is given in explicit recognition of the fact that the retardations present near the end of stage 1 (or in stage 2) are apt to be considerably different than those near its inception. *x_w_* will thus be used to deal approximately with the retardations in the region where the crystallization is ascribable to concurrent nucleation and growth, i.e., nearly up to the point of massive impingement at *x_m_.* On no account is it intended to convey that *x_w_* is, generally speaking, the true limiting degree of crystallinity in the system. The true limiting degree of crystallinity would by definition be an equilibrium state, undoubtedly only difficulty achievable in the type of homopolymer under consideration, whereas *x_w_*, as defined here, is determined by geometric and kinetic factors. The connection between *x_w_* and the pseudoequilibrium degree of crystallinity, *x_m_*, will be brought out shortly, but it is sufficient at this point to indicate that these two quantities will not generally be precisely the same on account of the expected differences in the retardations in the different portions of stage 1.

In this situation it is assumed that

$$dM_{c}=dM_{c}^{\prime }(1-x/x_{w}),$$ (A–1)

i.e., the actual increment of mass transformed in a time *dt* is proportional to the increment of mass transformed on a free growth basis times the mass fraction of (apparently) crystallizable polymer remaining. However, in any real case the possibility must be recognized that higher order terms in χ/χ*_w_* may exist that lead to deviations from the simple first-order rate law represented by eq (A–1). In such cases, χ*_w_ as defined by eq* (A–1) *will vary with* χ. This is in fact the type of result obtained by Lauritzen [16], who rigorously treated a model where rod-like objects born at *t*=0 at random positions and orientations in space grew lengthwise until they impinged on other growing rods. In a number of cases of interest studied by Lauritzen, χ*_w_* tended to start at a value of χ*_w_*(*_i_*)≅0.43 and then drop toward a value of χ*_m_* as the crystallization progressed. χ*_m_* depends on the scaling factor $n_{0}{\bar{r}}^{3}$. Here *n*_0_ is the number of nuclei per unit volume at *t*=0, and $\bar{r}$ the radius of each growth center. Lauritzen’s calculations provide a theoretical justification for the use of eq (4) or (A–4) with χ*_w_* being considered as an approximately constant and mean value of the apparent limiting degree of crystallinity in stage 1. It should be recognized that the χ*_w_*(*i*) and χ*_m_* values computed from Lauritzen’s theory may be somewhat too low to apply exactly in some real cases, since all impingements may not actually be effective in stopping growth. In cases where $n_{0}{\bar{r}}^{3}$ is such that χ*_m_* ≅ χ*_w_*(*_i_*), the mean value of χ*_w_* may be quite close to χ*_m_*, but a drop of χ*_w_* with increasing χ is commonly to be expected. In the experimentally accessible region, the mean value of χ*_w_* may generally be expected to lie in the range 0<χ*_w_*≤1. The calculations indicate that, barring certain effects such as markedly conical growth, χ*_w_* may in practice safely be assumed to lie between unity and somewhat below χ*_m_.* A value of χ*_w_*>1 is not excluded at very low χ, since here the crystals may be truly independent, and consume the liquid at the free growth rate, so that $dM_{c}≅dM_{c}^{\prime }$ or χ*_w_*>>1. However, such a contingency will not ordinarily arise in practice. Despite its limitations, eq (A–1) may be regarded as an approximate but adequate way of introducing the effect of retardations in the region of superposition. Equation (A–1) is certain to fail near and above χ*_m_.*

Dividing both sides of eq (A–1) by *M*_0_, and remembering that *dM_c_/M*_0_=*d_x_*, there is obtained

$$\frac{dx}{1—\left(x/x_{w}\right)}=\frac{dM_{c}^{\prime }}{M_{0}},$$ (A–2)

which, on integration, yields

$$x=x_{w}\left[1—e^{-\left(1/x_{w}\right)\left(M_{c}^{\prime }/M_{0}\right)}\right].$$ (A–3)

The quantity $M_{c}^{\prime }/M_{0}$is the mass fraction transformed on a free-growth basis, *x*′, and is readily calculated for various geometries of growth and types of nucleation. It generally takes the form *Zt^n^* (see below). Therefore eq (A–3) leads to

$$x=x_{w}\left[1—e^{-\left(1/x_{w}\right)Zt^{n}}\right].$$ (A–4)

### 9.2. Derivation of Equation (5)

Expand the exponential term in eq (A–4) to obtain

$$x=Zt^{n}—\left(1/2\right)\left(Z^{2}/x_{w}\right)t^{2n}+\ldots .$$ (A–5)

Now differentiate with respect to *t* to get

$$dx/dt=nZt^{n-1}—n\left(Z^{2}/x_{w}\right)t^{2n-1}+\ldots ,$$ (A–6)

and then divide by eq (A–5) and rearrange to find

$$n=\left(t/x\right)\left(dx/dt\right)\left[1+\left(1/2\right)\left(Z/x_{w}\right)t^{n}+\ldots \right].$$ (A–7)

*Z* must now be eliminated. Using eq (A–5) to find *Z*=*xt*^−^*^n^* (to the required accuracy only the first term need be retained), there is obtained

$$n=\left(t/x\right)\left(dx/dt\right)\left[1+\left(1/2\right)\left(x/x_{w}\right)+\ldots \right].$$ (A–8)

### 9.3. Free Growth Rate of Crystallites Born at the Same Time (Heterogeneous or Seed Crystal Nucleation)

There are *n*_0_ growth centers per unit volume in the system, and all are active at *t*=0. Then from the general formula [5] for the free growth rate, we have

$$x^{\prime }=\frac{M_{c}^{\prime }}{M_{0}}=n_{0}\left({\bar{V}}_{l}/{\bar{V}}_{c}\right)v^{\prime }\left(t,0\right),$$ (A–9)

where *v′(t*,0) is the volume of a given crystallite at time *t.*

#### One-dimensional Growth

Let $\bar{r}$ be the radius of the rod, which is taken to be of circular crosssection. Define *G*=*dl/dt* as the rate of lengthwise growth of the rod. *G* is taken to be constant at a given temperature (lineal growth). Then $v^{\prime }\left(t,0\right)=\pi {\bar{r}}^{2}Gt$, so that

$$\begin{array}{c} \frac{M_{c}^{\prime }}{M_{0}}=n_{0}\left({\bar{V}}_{l}/{\bar{V}}_{c}\right)\pi {\bar{r}}^{2}Gt=Z_{1}t.\\ \left(n=1\right) \end{array}$$ (A–10)

#### Two-dimensional Growth

If *l_d_* is the thickness of a disk growing radially at a rate *G_r_*=*dr/dt*, and *r* is the radius, then *v′*(*t*,0),= *πl_d_G*^2^*t*^2^. Therefore,

$$\begin{array}{c} \frac{M_{c}^{\prime }}{M_{0}}=n_{0}\left({\bar{V}}_{l}/{\bar{V}}_{c}\right)\pi l_{d}G_{r}^{2}t^{2}=Z_{2}t^{2}.\\ \left(n=2\right) \end{array}$$ (A–11)

### 9.4. Free Growth Rate of Crystallites Born at Later and Later Dates (Homogeneous or Pseudohomogeneous Nucleation)

For objects born at uniformly later and later times [5],

$$x\prime =\frac{M_{c}^{\prime }}{M_{0}}=\left({\bar{V}}_{l}/{\bar{V}}_{c}\right)\int_{0}^{t}Iv\prime \left(t,\tau \right)d\tau$$ (A–12)

*I* is the rate of injection of nuclei per unit volume, *τ* the birth time of a particle, where *τ≤t*, and *v*′(*t,τ*) is the volume at time *t* of a particle born at *τ*. The quantity *Idτ* represents the number of nuclei generated in a time *dτ*.

#### One-dimensional Growth

A nucleus born at *τ* occupies a volume $\pi {\bar{r}}^{2}G(t-\tau )$, since *t–τ* is the time it has grown. Hence, for constant *I* and *G*,

$$\begin{array}{l} \frac{M_{c}^{\prime }}{M_{0}}=({\bar{V}}_{l}/{\bar{V}}_{c})\pi r^{2}IG\int_{0}^{t}(t-\tau )d\tau \\ =({\bar{V}}_{l}/{\bar{V}}_{c})(\pi {\bar{r}}^{2}/2)IGt^{2}=Z_{2}t^{2}.\\ \left(n=2\right) \end{array}$$ (A–13)
